# Oxytocin Alleviates Colitis and Colitis-Associated Colorectal Tumorigenesis via Noncanonical Fucosylation

**DOI:** 10.34133/research.0407

**Published:** 2024-07-08

**Authors:** Xia Wang, Dawei Chen, Mengnan Guo, Yao Ning, Mingze Geng, Jing Guo, Jiahui Gao, Dong Zhao, Yupeng Zhang, Qianpeng Li, Lixiang Li, Shiyang Li, Yanqing Li, Xiaoran Xie, Xiuli Zuo, Jingxin Li

**Affiliations:** ^1^ Department of Gastroenterology, Qilu Hospital of Shandong University, Jinan, Shandong 250012, China.; ^2^ Laboratory of Translational Gastroenterology, Qilu Hospital of Shandong University, Jinan, Shandong 250012, China.; ^3^ Shandong Provincial Clinical Research Center for Digestive Disease, Jinan, Shandong 250012, China.; ^4^ Department of Physiology, School of Basic Medical Sciences, Cheeloo College of Medicine, Shandong University, Jinan, Shandong 250012, China.; ^5^ Department of Molecular Plant Biology, Norwegian Institute of Bioeconomy Research, Ås 1430, Norway.; ^6^ Department of Hematology, Weifang People’s Hospital, Weifang, Shandong 261000, China.; ^7^ Advanced Medical Research Institute, Shandong University, Jinan, Shandong 250012, China.

## Abstract

Colon cancer is increasing worldwide and is commonly regarded as hormone independent, yet recent reports have implicated sex hormones in its development. Nevertheless, the role of hormones from the hypothalamus–hypophysis axis in colitis-associated colorectal cancer (CAC) remains uncertain. In this study, we observed a significant reduction in the expression of the oxytocin receptor (OXTR) in colon samples from both patient with colitis and patient with CAC. To investigate further, we generated mice with an intestinal-epithelium-cell-specific knockout of OXTR. These mice exhibited markedly increased susceptibility to dextran-sulfate-sodium-induced colitis and dextran sulfate sodium/azoxymethane-induced CAC compared to wild-type mice. Our findings indicate that OXTR depletion impaired the inner mucus of the colon epithelium. Mechanistically, oxytocin was found to regulate Mucin 2 maturation through β_1_-3-*N*-acetylglucosaminyltransferase 7 (B3GNT7)-mediated fucosylation. Interestingly, we observed a positive correlation between B3GNT7 expression and OXTR expression in human colitis and CAC colon samples. Moreover, the simultaneous activations of OXTR and fucosylation by l-fucose significantly alleviated tumor burden. Hence, our study unveils oxytocin’s promising potential as an affordable and effective therapeutic intervention for individuals affected by colitis and CAC.

## Introduction

Inflammatory bowel disease (IBD), consisting of ulcerative colitis (UC) and Crohn’s disease (CD), poses a significant global public health challenge [1]. These conditions lead to debilitating gastrointestinal (GI) symptoms and progressive inflammation, resulting in irreversible damage to the GI tract and an escalated risk of colitis-associated colorectal cancer (CAC). The incidence of CAC is approximately 20% in UC and 8% in CD [2]. Therefore, understanding the pathogenesis of IBD and its association with CAC has important implications for the management of patients with IBD.

The mucus layer, acting as the primary barrier between intestinal bacteria and intestinal epithelial cells (IECs) [3], plays a critical role in the pathogenesis of IBD [4]. Mucin 2 (MUC2), synthesized by intestinal goblet cells (GCs), is a key component of the mucus layer [5]. Muc2-deficient mice spontaneously develop colitis and adenoma within the small and large intestine [5]*.* In addition, MUC2 is highly glycosylated, with O-linked glycans attached to serine or threonine residues having protective roles against intestinal inflammation and tumorigenesis [6]. However, the molecular mechanisms underlying the role of MUC2 in CAC remain unclear.

Protein glycosylation is a posttranslational modification that affects many biological processes such as cell adhesion, proliferation, and differentiation [7–9]. Fucosylation, one of the most common forms of glycosylation in mucins [10,11], links fucose to oligosaccharides and proteins. Fucosylation is constructed by fucosyltransferases (FUTs), guanosine diphosphate–fucose transporters, and guanosine diphosphate–fucose synthetic enzymes using the substrate fucose [10]. Aberrant fucosylglycans have been observed in various diseases. For example, fucosylation of mucins increases mucus viscoelasticity and its resistance to shear stress [6]. Gut microbiota promotes UC in intestinal-epithelium-specific FUT2 deficiency [12]. FUT8 modifies the biophysical properties of mucus and facilitates bacterial–epithelial interactions leading to UC progression [13]. However, it is still unknown whether deficiency of fucosylation is causative of a pathogenic role in UC and CAC development.

The GI hormones are well known to regulate gut mucosal physiology via their specific receptors [14]. Recently, the functions of non-GI hormones in colon pathology have begun to show the interplay between the non-GI hormones and colonic growth [14]. In this report, we uncovered that the role of oxytocin (OXT), a neurohypophysial hormone and neuropeptide primarily synthesized by the hypothalamus and released by the pituitary gland [15–17], exerts its effects through the OXT receptor (OXTR). OXTR belongs to the G-protein-coupled receptor class A/rhodopsin family and plays a pivotal role in social bonding, stress response, maternal behavior, sexual activity, uterus contraction, milk ejection, and cancer [16–18]. Emerging evidence suggests the presence of OXT/OXTR in the GI system [19–21] and reports the important roles of OXT in GI motility, inflammation, macromolecular permeability, and mucosal maintenance in mice [22]. However, the role of OXT/OXTR in IECs remains poorly understood, necessitating further mechanistic investigations in GI diseases.

This study aimed to uncover the essential function of OXT signaling in colonic carcinogenesis and colitis. We found that the deficiency of OXTR in IECs may render them more susceptible to CAC and UC. Mechanistically, we investigated the impact of OXT/OXTR on the mucosal barrier function by examining the transcriptional activation of β_1_-3-*N*-acetylglucosaminyltransferase 7 (B3gnt7) and the induction of α_1_-3-fucosylation of MUC2. Our findings provide valuable insights into a novel diagnostic indicator for CAC and colitis, contributing to a better understanding and management of these conditions.

## Results

### IEC ablation of OXTR sensitizes mice to CAC

**Fig. 1.**
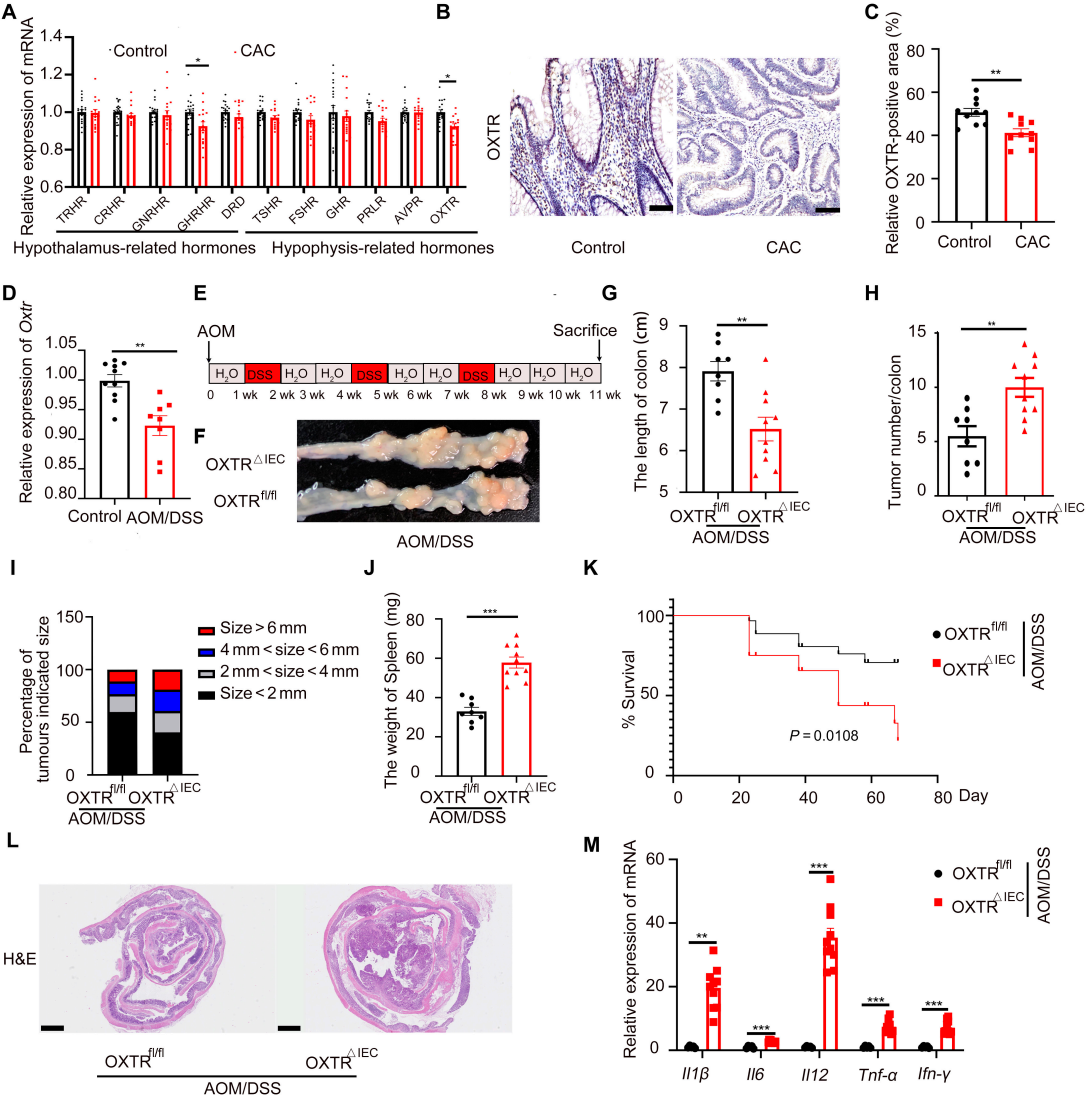
Ablation of OXTR in IECs promotes the development and progression of colitis-associated cancer. (A) Analysis of hypothalamus–hypophysis hormone receptors gene expression in colonic tissue from normal and patients with CAC. TRHR, thyrotropin-releasing hormone receptor; CRHR, thyrotropin-releasing hormone receptor; GNRHR, gonadotropin-releasing hormone receptor; DRD, dopamine receptor D; TSHR, thyroid stimulating hormone receptor; FSHR, thyroid stimulating hormone receptor; GHR, growth hormone receptor; PRLR, prolactin receptor; AVPR, arginine vasopressin receptor. (GSE37283 and GSE47908). (B) Representative images of OXTR immunohistochemical staining in human adjacent (left) and CAC (right) tissue. Scale bars, 100 μm. (C) The quantifications of OXTR-positive area. measured by ImageJ. Two biopsies were quantified per patient. *n* = 5 patients. (D) OXTR gene expression in colon tissue from AOM/DSS-induced cancer model. (GSE113002). (E to M) OXTR^fl/fl^ and OXTR^△IEC^ mice were treated with AOM and 3 cycles of 2% DSS to induce inflammation-driven colorectal cancer. (E) Flow chart of the induction procedure. (F) Representative photos of the colons. (G) Colon length of OXTR^fl/fl^ (*n* = 8) and OXTR^△IEC^ (*n* = 10) mice. (H) Number of tumors in OXTR^fl/fl^ (*n* = 8) and OXTR^△IEC^ (*n* = 10) colon. (I) The percentage of different tumor diameters in OXTR^fl/fl^ (*n* = 8) and OXTR^△IEC^ (*n* = 10) mice. (J) Spleen weight of OXTR^fl/fl^ (*n* = 8) and OXTR^△IEC^ (*n* = 10) mice. (K) Survival curve assessed by log-rank (Mantel–Cox) test. (L) Representative H&E-stained colon sections in OXTR^fl/fl^ (*n* = 8) and OXTR^△IEC^ (*n* = 10) mice. Scale bars, 1mm. (M) Relative inflammatory cytokine mRNA levels in OXTR^fl/fl^ (*n* = 8) and OXTR^△IEC^ (*n* = 10) colons were measured by real-time PCR. Data are presented as the means ± SEM. *P < 0.5, **P < 0.1, and ***P < 0.01. *P* values were determined using (A, K, and M) 2-way ANOVA or (C, D, and G to J) unpaired 2-tailed Student’s *t* test.

Initially, we examined the expression of relevant receptors of hormones released by the hypothalamus–hypophysis axis, which controls the production and secretion of hormones through the pituitary portal vein system [23,24], in CAC using the Gene Expression Omnibus (GEO) database and found that the expression of growth hormone releasing hormone receptor (GHRHR) and OXTR genes was decreased (Fig. 1A). Since the role of GHRHR in CAC is well established [24], we focused on OXT. We observed decreased expression of OXTR in patients with CAC compared to adjacent healthy tissue (Fig. 1B and C). Consistent with these findings, the expression of OXTR was also weakened in a mouse colon cancer model induced by azoxymethane (AOM)/dextran sulfate sodium (DSS) (Fig. 1D and Fig. S1A). To examine the functional role of OXTR in CAC, we established OXTR-deficient (OXTR^△IEC^) mice (Fig. S1B and C) and performed an AOM/DSS model (Fig. 1E). Following the AOM/DSS protocol, OXTR^△IEC^ mice exhibited significantly increased tumor burden compared to OXTR^flox/flox^ (OXTR^fl/fl^) mice (Fig. 1F). Statistical analysis revealed that OXTR^△IEC^ mice had a shorter colon length (Fig. 1G), a higher number of tumors (Fig. 1H), a wider tumor area and tumor load (Fig. 1I), and increased spleen weight (Fig. 1J). The survival time of OXTR^△IEC^ mice was significantly shorter (Fig. 1K). Hematoxylin and eosin (H&E) staining demonstrated substantial tumor areas, more colon injury, and immune cell infiltration in the colon tissues of OXTR^△IEC^ (Fig. 1L). Moreover, reverse transcription quantitative polymerase chain reaction (RT-qPCR) analysis showed increased expression of inflammatory factors (Fig. 1M). Overall, these results indicate that OXTR deficiency in IECs exacerbates AOM/DSS-induced CAC progression.

### OXTR deficiency in IEC facilitates that CAC progress depends on inflammation

**Fig. 2.**
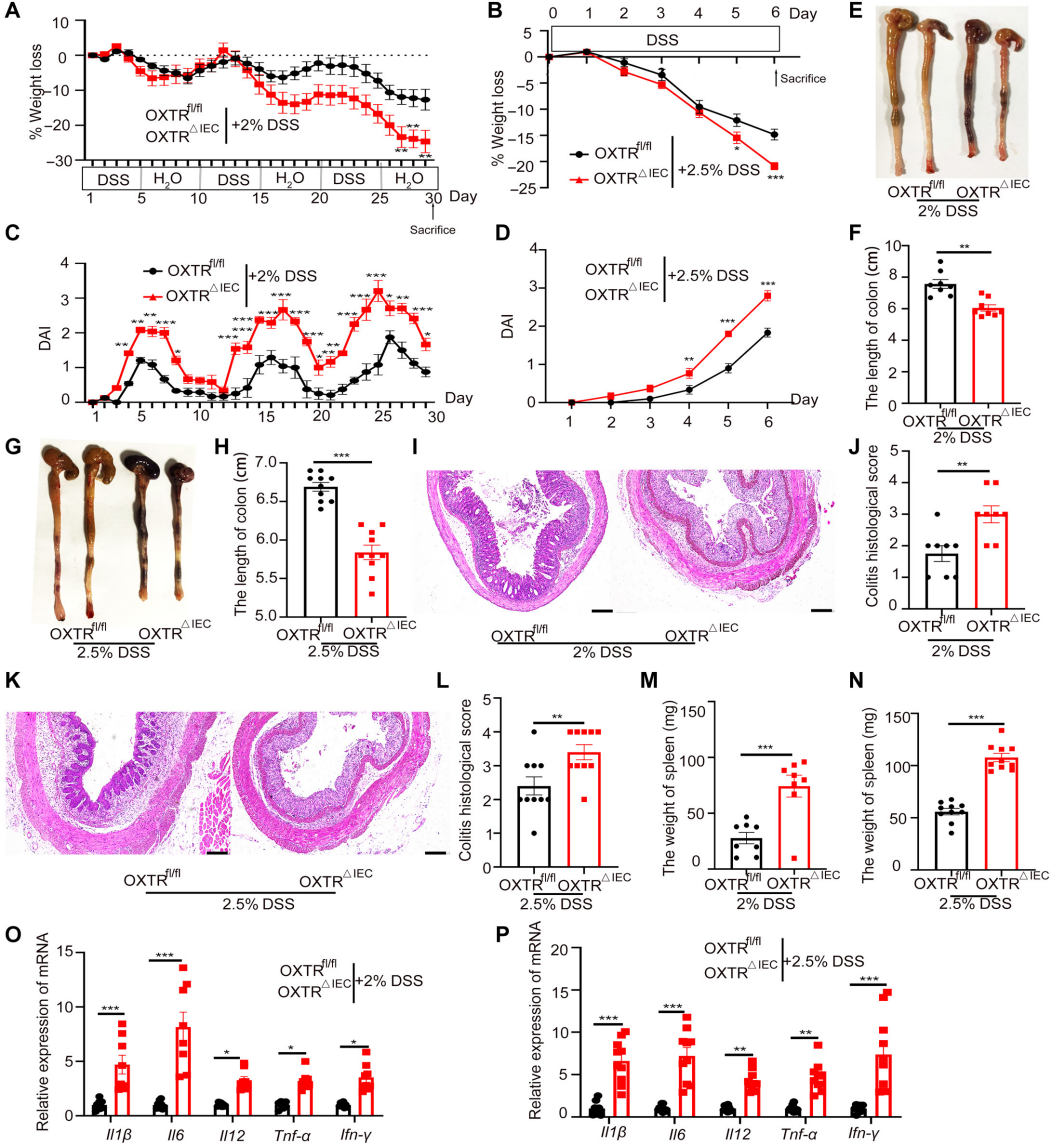
OXTR IEC-deficient mice develop severe DSS-induced colitis. OXTR^fl/fl^ and OXTR^△IEC^ mice were treated with 2% DSS (*n* = 8) or 2.5% DSS (*n* = 10) to induce chronic colitis and severe colitis. (A and B) Body weights were measured daily and are depicted as a percentage of initial body weight in 2% DSS model (A) or 2.5% DSS model (B). (C and D) The disease activity index (DAI) was scored in 2% DSS model (C) or 2.5% DSS model (D). (E to H) Representative photos of the colons and statistical analysis of colon length in 2% DSS model (E and F) or 2.5% DSS model (G and H). (I to L) Representative H&E-stained images and semiquantitative scoring of histopathology in chronic colitis (I and J) or severe colitis tissue (K and L). Scale bars, 100 μm. (M and N) Statistical analysis of spleen weight in 2% DSS model (M) or 2.5% DSS model (N). (O and P) RT-qPCR analysis of the relative expression of inflammatory factors in chronic colitis (O) or severe colitis (P) tissues. Data are presented as the means ± SEM. *P < 0.5, **P < 0.1, and ***P < 0.01. *P* values were determined using (A to D, O, and P) 2-way ANOVA or (F, H, J, and L to N) unpaired 2-tailed Student’s *t* test.

The AOM/DSS-mediated primary cancer model involves synergistic effects of AOM-induced mutation and DSS-induced inflammation in CAC development [25]. To determine whether AOM or inflammation is necessary for OXTR to suppress CAC, we evaluated a colitis-independent colon cancer model (Fig. S2A). Our results showed no significant difference in tumor incidence including colon length, tumor burden, spleen weight, histological characteristics, or inflammation levels between the 2 groups (Fig. S2B to F). In addition, we administered 2% DSS or 2.5% DSS to induce chronic colitis and acute colitis. Compared with OXTR^fl/fl^ mice, OXTR^△IEC^ mice exhibited exacerbated colitis, with more significant body weight reduction (Fig. 2A and B), higher stool score and bleeding scores (Fig. 2C and D), shorter colon length (Fig. 2E to H), as well as enlarged histologic mucosal damage (Fig. 2I to L), larger spleen size (Fig. 2M and N), and higher levels of inflammatory cytokines (Fig. 2O and P). Importantly, the absence of OXTR does not trigger intestinal inflammation in a steady state (Fig. S3). These findings indicate that OXT suppresses the development of colon cancer by inhibiting inflammation.

### IEC-specific ablation of OXTR impairs the levels of MUC2 via B3GNT7

To understand how OXT signaling modulates the pathogenesis of DSS-induced colitis, we evaluated the expression levels of colonic barrier proteins in OXTR^fl/fl^ and OXTR^△IEC^ IECs with or without DSS treatment. We found that the absence of OXTR did not affect the mRNA (Fig. S4) and protein levels of the tight junction but did affect the protein levels of the mucin MUC2, the dominant component of the inner mucus layer (Figs. S5A to C and S9A and B) [26–28]. As the inner mucus layer, a physical barrier, prevents excessive inflammation by separating microbes [28], we examined its integrity. Our results showed that exposure to DSS led to a considerable decline in the thickness of the inner mucus layer (Fig. S5D and E). Importantly, compared to OXTR^fl/fl^, the inner mucus layer was extensively damaged in OXTR^△IEC^ mice even under physiological conditions and near-complete disappearance in DSS-treated OXTR^△IEC^ mice (Fig. S5D and E). The inner mucus layer is primarily produced by colonic GCs, specialized cells located in the epithelium [26]. Interestingly, we found that the differentiation of GCs was comparable between OXTR-deficient and OXTR^fl/fl^ mice (Fig. S5, F and G), although the expression of MUC2 was decreased (Fig. S5, H and I). Notably, OXT up-regulated MUC2 expression in LS174T cells and OXTR^fl/fl^ colonic organoids, while the inhibition of OXTR with L-368,899 abolished the effect of OXT (Figs. S6A to C and S9C and D). Furthermore, in OXTR-deficient organoids, OXT had no impact (Figs. S6A to C and S9D). Collectively, these findings suggest that OXT/OXTR regulates the integrity of the inner mucus layer by modulating the expression of MUC2.

**Fig. 3.**
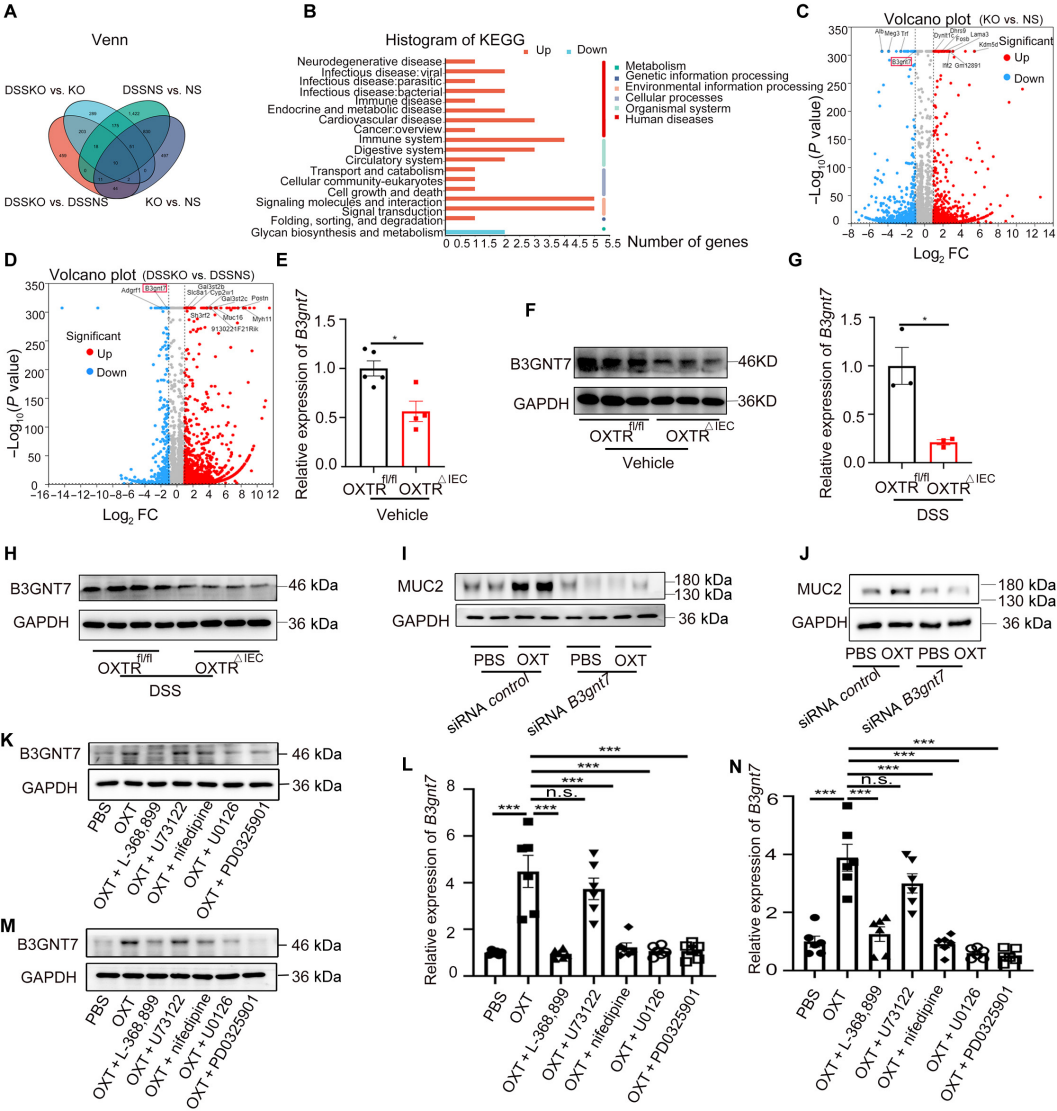
OXT regulates MUC2 expression via B3GNT7. (A) A Venn diagram was plotted to show the intersected genes from 4 groups, NS (OXTR^fl/fl^) and KO (OXTR^△IEC^), and 10 common genes were screened out. (B) The Kyoto Encyclopedia of Genes and Genomes (KEGG) pathways for the up-regulated mRNAs and down-regulated mRNAs. (C and D) Fold change (FC) (log_2_) in gene expression of in the IECs from OXTR^fl/fl^ (*n* = 3) and OXTR^△IEC^ (*n* = 3) mice treated with or without DSS, plotted against significance [−log_10_(*P* value)]. Up-regulated genes (*P* < 0.05) are represented in red, and down-regulated genes are labeled in blue. (E) RT-qPCR analysis of the expression of B3GNT7 in the IECs from OXTR^fl/fl^ and OXTR^△IEC^ mice. *n* = 5. (F) Western blot analysis of the expression of B3GNT7 in the IECs from OXTR^fl/fl^ and OXTR^△IEC^ mice. *n* = 5. (G) RT-qPCR analysis of the expression of B3GNT7 in the IECs from OXTR^fl/fl^ and OXTR^△IEC^ mice treated with 2.5% DSS. *n* = 3. (H) Western blot analysis of the expression of B3GNT7 in the IECs from OXTR^fl/fl^ and OXTR^△IEC^ mice treated with 2.5% DSS. *n* = 5. (I and J) Western blot analysis of the MUC2 level in LS174T cells (I) and colonic organoids (J) treated with OXT (1 μmol) or siRNA (50 nmol). *n* = 5. (K and M) Western blot analysis of the expression of B3GNT7 and glyceraldehyde-3-phosphate dehydrogenase (GAPDH) in colonic organoids (K) and LS174T cells (M). PBS, phosphate-buffered saline. *n* = 6. (L and N) RT-qPCR analysis of the expression of B3gnt7 in colonic organoids (L) and LS174T cells (N) treated with OXT (1 μmol), L-368,899 (1 μmol), U73122 (1 μmol), nifedipine (1 μmol), U0126 (20 μmol), PD0325901 (1 μmol), or vehicle control. *n* = 6. Data are presented as the means ± SEM. *P < 0.5 and ***P < 0.01. *P* values were determined using (E and G) an unpaired 2-tailed Student’s *t* test or (L and N) one-way ANOVA. n.s., not significant.

To investigate the effect of OXT signaling on MUC2 expression, we conducted RNA sequencing analysis on OXTR^fl/fl^ and OXTR^△IEC^ IECs under physiological and pathological conditions. As detailed in Fig. 3A, our analysis identified 10 genes that were consistently down-regulated across 4 experimental groups (KO versus NS, DSSKO versus DSSNS, NS versus DSSNS, and KO versus DSSKO), all of which are implicated in glycan biosynthesis and metabolism, as illustrated in Fig. 3B. Among these down-regulated genes, B3gnt7 was particularly notable for its involvement in the O-linked glycosylation of mucins, a key process in colon physiology (Fig. 3C and D) [29]. RT-qPCR and Western blot analysis confirmed the decreased expression of B3GNT7 in OXTR^△IEC^ colonic epithelial cells (Fig. 3E to H and Fig. S9E and F). In addition, incubation with OXT up-regulated B3GNT7 expression in LS174T cells and colonic organoids (Figs. S6D to G and S9G and H). Moreover, down-regulation of B3gnt7 using small interfering RNA (siRNA) in LS174T cells (Figs. S6H and I and S9I) and colonic organoids (Figs. S6J and K and S9J) inhibited the OXT-dependent increase in MUC2 expression (Fig. 3I and J and Fig. S9K and L). It is established that OXT can induce Ca^2+^ influx [30] and activate the mitogen-activated protein kinase kinase (MEK)/extracellular-signal-regulated kinase (ERK)/ribosomal protein S6 kinase signaling pathway [31], which are critical for various cellular functions. To elucidate the specific pathways through which OXT influences B3gnt7 expression, we used several pharmacological inhibitors in our experimental design. Our results demonstrate that the OXTR antagonist L-368,899, the L-type Ca^2+^ channel blocker nifedipine, and the MEK/ERK inhibitors U0126 or PD0325901 significantly inhibited B3GNT7 expression in colonic organoids (Fig. 3K and L and Fig. S9M) and LS174T cells (Fig. 3M to N and Fig. S9N). Conversely, inhibition with U73122, a phospholipase-C-dependent Ca^2+^ channel inhibitor, did not affect the OXT-enhanced B3gnt7 production. These findings suggest that the regulation of MUC2 by OXT likely occurs through the L-type Ca^2+^ channel and the MEK/ERK/B3GNT7 pathways, rather than through phospholipase-C-dependent Ca^2+^ signaling.

### OXT/OXTR induces MUC2 α_1_-3-fucosylation for protection against colitis

**Fig. 4.**
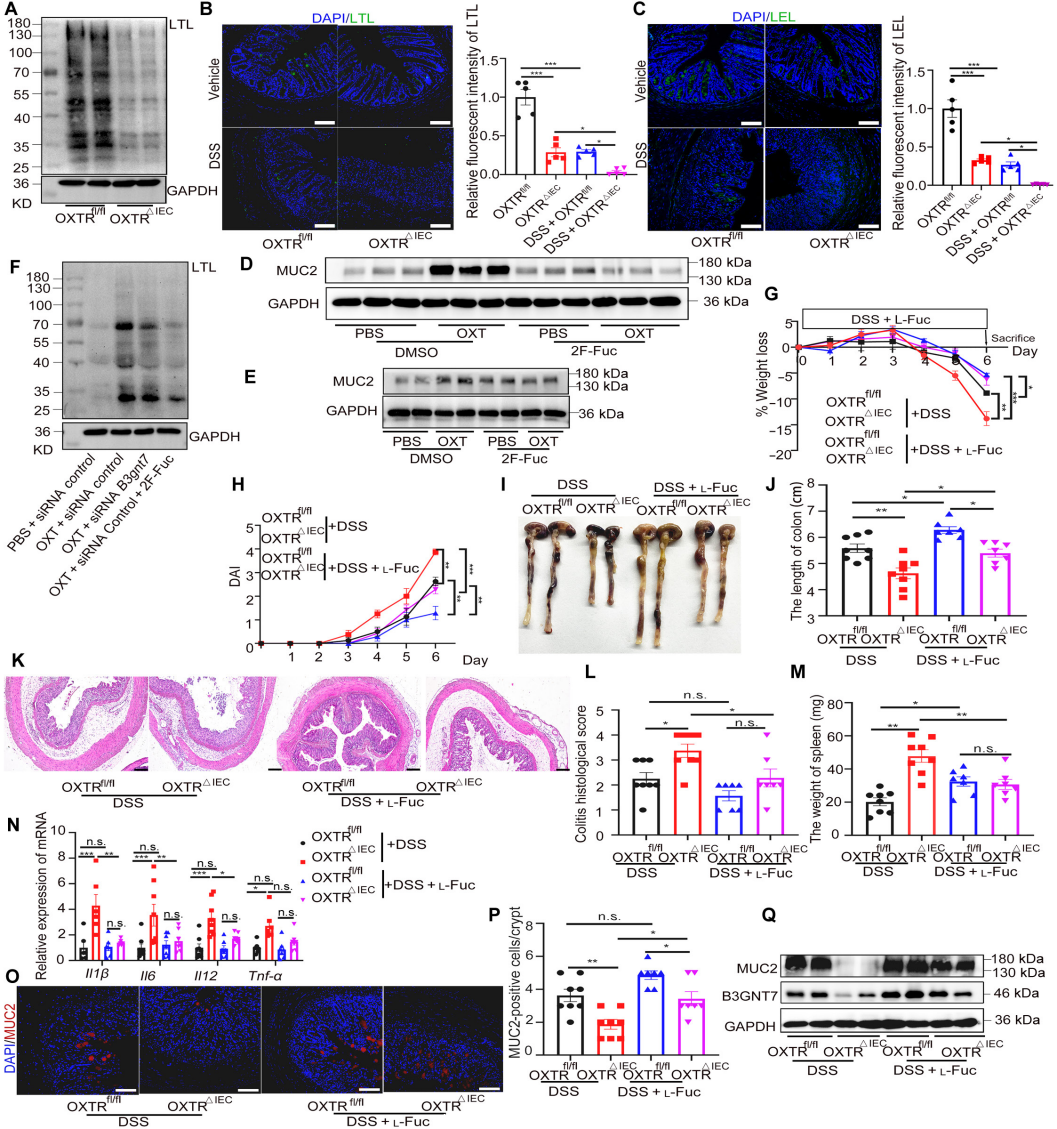
Fucosylation is responsible for OXT-mediated protective effect in DSS-induced acute colitis. (A) Western blot analysis of the LTL level in the IECs from OXTR^fl/fl^ and OXTR^△IEC^ mice. *n* = 5. (B) Immunofluorescence staining and quantitative analyses of LTL (green) and nuclei [4′,6-diamidino-2-phenylindole (DAPI), blue]. Scale bars, 100 μm. *n* = 5. (C) Immunofluorescence staining and quantitative analyses of LEL (green) and nuclei (DAPI, blue). Scale bars, 100 μm. *n* = 5. (D and E) Western blot analysis of the MUC2 level in LS174T cells (D) and colonic organoids (E) treated with OXT (1 μmol) or 2F-Fuc (200 μmol). *n* = 5. (F) Western blot analysis of the LTL level in LS174T cells treated with siRNA, OXT, or 2F-Fuc. *n* = 5. (G to Q) OXTR^fl/fl^ and OXTR^△IEC^ mice were treated with 2.5% DSS (*n* = 8) or 200 mM l-fucose (l-Fuc) (*n* = 7). (G) Body weights were measured daily and are depicted as a percentage of initial body weight. (H) DAI score. (I) Representative photos of the colons. (J) Statistical analysis of colon length. (K) Representative H&E-stained images. Scale bars, 100 μm. (L) Histologic injury score. (M) Statistical analysis of spleen weight. (N) RT-qPCR analysis of the relative expression of inflammatory factors in colon tissues. (O) Immunofluorescence staining of Muc2 (red) and nuclei (DAPI, blue). Scale bars, 100 μm. (P) The number of MUC2-positive cells were counted randomly in crypts for each mouse. (Q) Western blot analysis of the MUC2, B3GNT7, and GAPDH levels in the IECs from OXTR^fl/fl^ and OXTR^△IEC^ mice treated with or without l-fucose. *n* = 5. Data are presented as the means ± SEM. *P < 0.5, **P < 0.1, and ***P < 0.01. *P* values were determined by using a 2-way ANOVA.

B3GNT7, which is involved in the combination of poly-*N*-acetyllactosamine chains and triggers α_1_-3-fucosylation of glycoproteins in IECs [29,32], has been exhibited to inhibit the metastasis of colon cancer cells. Since OXT facilitates the expression of B3GNT7 (Fig. 3), we explored whether OXT also promotes fucosylation in IECs. We evaluated the overall fucosylation status of glycoproteins using fucose-recognizing lectins in IEC lysates. The results showed attenuated binding of lotus tetragonolobus lectin (LTL), which characteristically identifies α_1_-3-linked fucose [32,33], in OXTR-deficient IECs (Fig. 4A and Fig. S9O). In addition, we compared fucosylation patterns in the colon tissues of OXTR^fl/fl^ and OXTR^△IEC^ mice using LTL staining and found reduced lectin binding in OXTR^△IEC^ mice (Fig. 4B). To understand the mechanisms underlying OXT-induced increased α_1_-3-fucosylation, we examined the expression levels of FUTs, B3GNTs, and glycosidases in IECs [32]. Interestingly, except for B3gnt7, the transcription levels of other enzymes remained unchanged (Fig. S7). Furthermore, we observed reduced binding of lycopersicon esculentum lectin (LEL), which recognizes *N*-acetylglucosamine (GlcNAc) residues in chitin and poly-*N*-acetyllactosamine chains [29,34], in the colon tissues of OXTR^△IEC^ mice (Fig. 4C).

The expression and O-glycosylation alterations of MUC2 mucins have been implicated in colorectal cancer [6]. To examine the effects of fucosylation regulated by OXT on LS174T cells and colonic organoids, we used 2-fluoroperacetyl-fucose (2F-Fuc), a metabolic inhibitor of fucosylation, and found that the up-regulation of MUC2 by OXT was inhibited in the presence of 2F-Fuc [29], indicating that increased MUC2 expression depends on fucosylated structures (Fig. 4D and E and Fig. S9P and Q). Moreover, OXT can up-regulate the level of fucosylation, and this effect can be inhibited when B3GNT7 expression is reduced, which further indicates that OXT can regulate fucosylation through B3GNT7 (Fig. 4F and Fig. S9R). To further investigate the role of fucosylation-mediated MUC2 in the maintenance of colitis, we tested the effect of l-fucose supplementation, an essential substrate of fucosylation [12], in a DSS-induced colitis model. Remarkably, OXTR^△IEC^ mice treated with l-fucose showed improved weight loss and disease activity index compared to those without l-fucose (Fig. 4G and H). In addition, DSS-induced colon shortening was significantly ameliorated in OXTR^fl/fl^ and OXTR^△IEC^ mice receiving l-fucose (Fig. 4I and J). Histological damage and the levels of inflammation were also reduced in l-fucose-treated mice, with less epithelial damage, crypt loss, spleen enlargement, and inflammatory factor content (Fig. 4K to N). Importantly, the treatment of OXTR-deficient mice with l-fucose led to a significant increase in colonic MUC2 and B3GNT7 levels (Fig. 4O to Q and Fig. S9S and T). These findings suggest that l-fucose treatment restores colonic MUC2 concentration in OXTR^△IEC^ mice and alleviates DSS-induced colitis.

### Treatment with OXT suppresses CAC development

Having established that mice with OXTR deficiency in intestinal epithelium exhibit more advanced colitis and colon cancer (Figs. 1 to 4), we further investigated the role of the OXT system in CAC through OXT supplementation (Fig. S8A). Mice treated with OXT exhibited longer colon lengths compared to control mice (Fig. S8B and C). Furthermore, OXT-treated mice developed significantly fewer and smaller tumors in the colon compared to OXTR^fl/fl^ mice (Fig. S8D and E). Statistical analysis confirmed reduced spleen size and prolonged survival time in OXT-treated mice (Fig. S8F and G). Histologically, OXTR^fl/fl^ mice treated with OXT showed reduced tumor areas, less colon injury, and immune cell infiltration (Fig. S8H). Moreover, the inflammation was suppressed in OXTR^fl/fl^ mice treated with OXT (Fig. S8I). Functionally, we confirmed that OXT administration significantly elevated the levels of B3GNT7, MUC2, and fucosylation in both the AOM/DSS (Figs. S8J and S9U) and the DSS (Figs. S8K and S9V) models. In addition, OXT enhanced the thickness of the colonic mucus layer in mice treated with 2.5% DSS (Fig. S8L). These findings underscore the protective role of OXT in colitis-associated colon cancer by MUC2 fucosylation.

### l-Fucose has synergistic effects with OXT treatment in CAC models

**Fig. 5.**
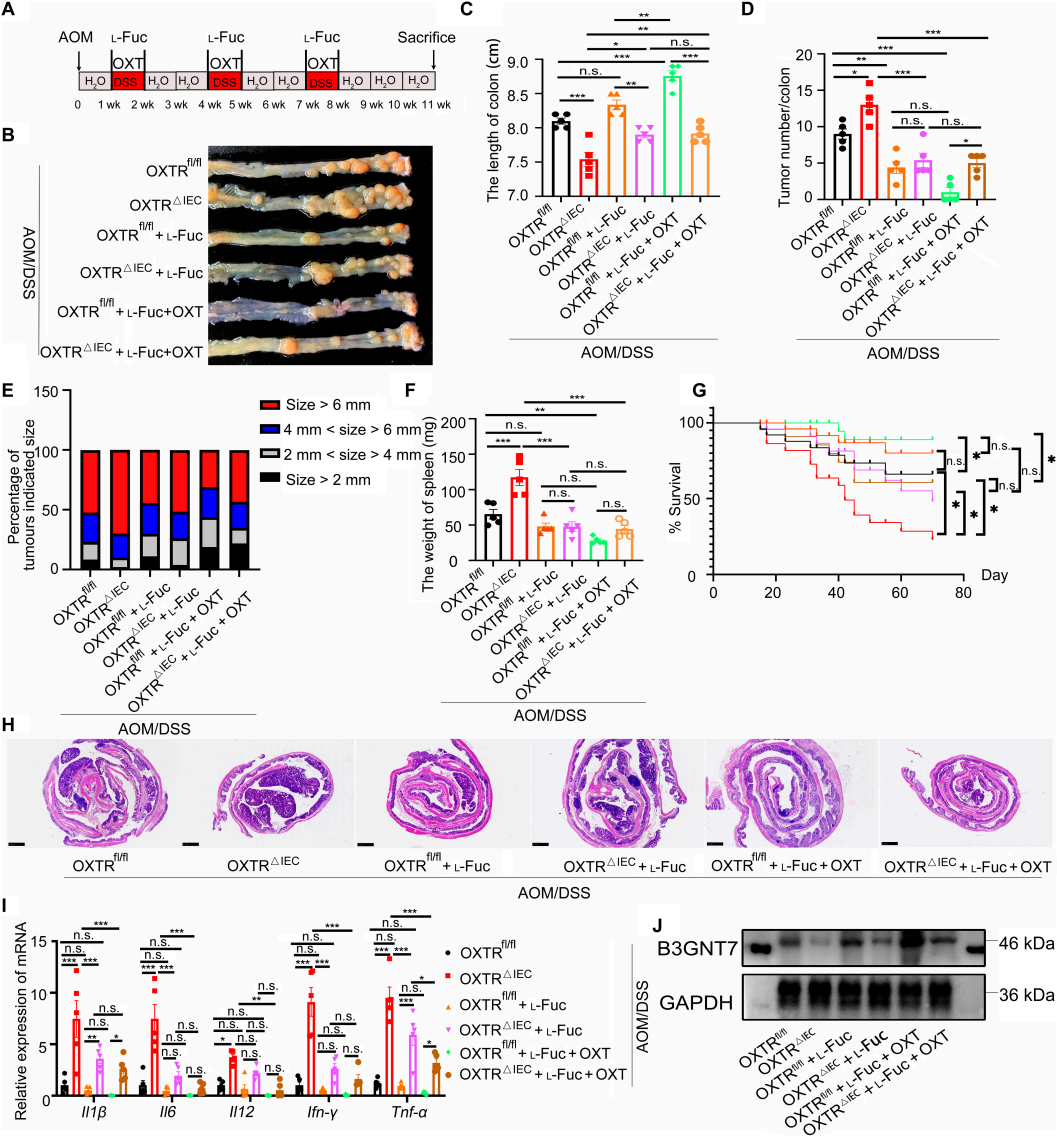
l-Fucose and OXT combination therapy inhibits the growth of colon carcinoma tumor. OXTR^fl/fl^ and OXTR^△IEC^ mice were treated with AOM and 3 cycles of 2% DSS, DSS + l-fucose, or DSS +l-fucose+ OXT to induce inflammation-driven colorectal cancer. (A) Schema of sample collection and analysis. (B) Photographs of representative tumor blocks collected from different treatment groups. (C) Colon length. *n* = 5. (D) Number of tumors in the colon. *n* = 5. (E) The percentage of tumors by different diameters. *n* = 5. (F) Statistical analysis of spleen weight. *n* = 5. (G) Survival curve assessed by log-rank (Mantel–Cox) test in OXTR^fl/fl^ (*n *= 15), OXTR^△IEC^ (*n* = 20), OXTR^fl/fl^ + l-fucose (*n *= 15),OXTR^△IEC^ + l-fucose (*n *= 16),OXTR^fl/fl ^+ l-fucose + OXT (*n *= 12), and OXTR^△IEC^ + l-fucose + OXT (*n *= 13) mice. (H) Representative H&E-stained colon sections. Scale bars, 1 mm. (I) Relative inflammatory cytokine mRNA levels in the colon were measured by real-time PCR. *n* = 5. (J) Western blot analysis of the B3GNT7 and GAPDH levels in OXTR^fl/fl^ and OXTR^△IEC^ mice treated with or without l-fucose and OXT. *n* = 5. Data are presented as the means ± SEM. *P < 0.5, **P < 0.1, and ***P < 0.01. *P* values were determined using (C to G) one-way ANOVA or (J) 2-way ANOVA.

Given that l-fucose protected OXTR^fl/fl^ and OXTR^△IEC^ mice from DSS colitis (Fig. 4), we next determined whether l-fucose could have a protective effect on the development of CAC (Fig. 5A). Mice receiving l-fucose showed longer colon lengths (Fig. 5B and C), reduced tumor burden (Fig. 5D to F), longer lifespan (Fig. 5G), and lower inflammation levels (Fig. 5H and I) compared with untreated mice, indicating that l-fucose alone may have single-agent activity, although all mice eventually attained a tumor burden. To extend and validate the effects of l-fucose and OXT, we treated mice with l-fucose in combination with OXT and found that the addition of OXT to l-fucose therapy resulted in significantly increased tumor shrinkage (Fig. 5A to J). However, OXT treatment showed improved tumor regression in OXTR^fl/fl^ mice compared with OXTR^△IEC^ mice, as indicated by reduced tumor burden (Fig. 5D and E) and increased survival (Fig. 5G), indicating possible OXT single-agent therapeutic activity, with 72% of mice receiving l-fucose alone cured compared with 83% of mice receiving OXT and l-fucose therapy in OXTR^fl/fl^ mice, whereas 58% of mice receiving l-fucose alone cured compared with 62% of mice receiving OXT and l-fucose therapy in OXTR^△IEC^ mice (Fig. 5G). Mechanically, we found that a combination administration of l-fucose and OXT significantly elevated the levels of B3GNT7 in both OXTR^fl/fl^ mice and OXTR^△IEC^ mice (Fig. 5J and Fig. S9W) models. Together, these results show the therapeutic benefit of l-fucose as monotherapy and in combination with OXT therapy.

### Decreased expression of B3GNT7 correlates with OXTR in human colitis and CAC

**Fig. 6.**
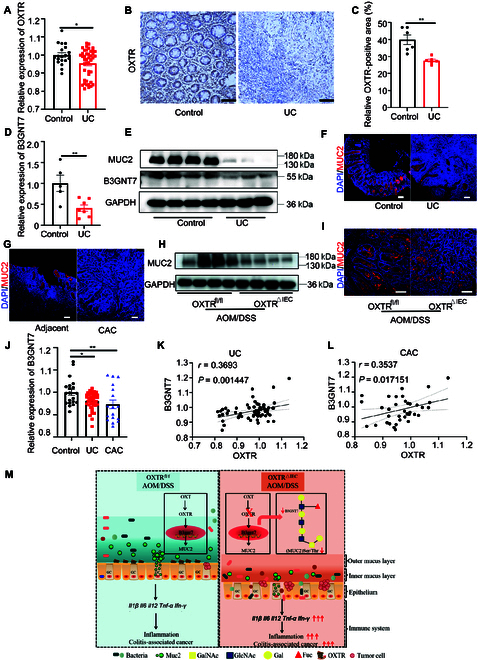
OXTR and B3GNT7 expressions in human colitis. (A) OXTR gene expression in human normal (control) and UC tissue. (GSE37283 and GSE47908). (B) Representative images of OXTR staining on human normal and UC tissue. Scale bars, 100 μm. (C) The quantifications of OXTR-positive area. *n* = 6. (D) RT-qPCR analysis of the relative expression of B3GNT7 in human normal (*n* = 5) and UC (*n* = 7) tissue. (E) Western blot analysis of the MUC2 and B3GNT7 levels in human normal (*n* = 4) and UC tissue (*n* = 3). (F) Immunofluorescence staining of Muc2 (red) and nuclei (DAPI, blue) in human normal (*n* = 5) and UC tissue (*n* = 5). Scale bars, 100 μm. (G) Immunofluorescence staining of Muc2 (red) and nuclei (DAPI, blue) in human adjacent (*n* = 3) and CAC tissue (*n* = 3). Scale bars, 100 μm. (H and I) Western blot analysis (H) and immunofluorescence staining (I) of MUC2 in OXTR^fl/fl^ and OXTR^△IEC^ mice treated with AOM/DSS. (*n* = 4). Scale bars, 100 μm. (J) B3GNT7 gene expression in human normal, UC, and CAC tissue. (GSE37283 and GSE47908). (K and L) Correlations between the expression of B3GNT7 and OXTR in human UC (K) and CAC (L). (M) Schematic model depicting the functions of intestinal OXTR in CAC. Data are presented as the means ± SEM. *P < 0.5 and **P < 0.1. *P* values were determined using (A, C, and D) unpaired 2-tailed Student’s *t* test or (J) 2-way ANOVA.

To understand the relevance of the decreased expression of OXTR and B3GNT7 in humans, we analyzed an independent cohort of patients with UC and found decreased expression of OXTR in colitis (Fig. 6A). Using patient samples, we observed reduced expression of OXTR in the colon tissues of patients with UC compared to normal controls (Fig. 6B and C). Consistent with the animal results, B3GNT7 and MUC2 (Fig. 6D to F and Fig. S9X) expression was significantly lower, and the expression level of FUTs (Fig. S7D) was unchanged in human colitis. Furthermore, we examined colon tissue and IECs from patients with CAC (Fig. 6G) and an AOM/DSS mouse model (Fig. 6H and I). We found reduced MUC2 expression in colitis-associated cancer (Fig. 6G to I and Fig. S9Y). In addition, in human patients with UC and patients with colitis-associated cancer, the expression of B3GNT7 was decreased and positively correlated with OXTR (Fig. 6J to L). These results indicate that the down-regulation of B3GNT7 is strongly associated with OXTR in human inflammation and colonic carcinogenesis.

## Discussion

In this study, we present compelling evidence supporting the involvement of OXT in the formation of colonic mucus and its protective role in colitis and CAC tumorigenesis. We demonstrate that the depletion of OXTR specifically in IECs contributes to the development of experimental colitis induced by DSS and CAC induced by a combination of DSS and AOM. To unravel the underlying molecular mechanisms, we elucidate that OXT orchestrates the maturation of colonic MUC2 proteins through B3GNT7-mediated fucosylation. Our study uncovers a novel regulatory pathway wherein OXT controls the fucosylation process crucial for the formation of colonic mucin. Remarkably, combination treatment with OXT and l-fucose ameliorates tumor burden in CAC development.

While OXT is widely recognized for its involvement in behaviors such as social bonding, reproduction, childbirth, and postpartum functions [17], we extend the understanding of its role in the GI system. Enteric nerves can release OXT and various cells in the intestine express OXTR [15,16,19,35], indicating its multifaceted functions beyond the traditional domains. Our previous studies have already demonstrated the beneficial effects of OXT in mitigating DSS-induced intestinal injury by inducing prostaglandin E2 release [36], as well as its regulatory roles in macrophage polarization [37], and dendritic cell tolerance [38] to alleviate DSS-induced colitis. Here, our findings provide an explanation that activation of OXT signaling in the colonic epithelium promoted protective mucin protein MUC2 maturation by B3GNT7-mediated fucosylation. MUC2 is the first defense layer of the colon and plays a vital role in colitis and CAC development [28]. Hormones can regulate plenty of gene expression through the activation of transcription factors. The OXT-involved posttranslational modification of MUC2 we identified may be a general way for hormones’ physiological functions.

Interestingly, prior research utilizing systematic bioinformatics analysis has suggested that high levels of OXTR mRNA are associated with a poor prognosis in colon cancer [18], implicating OXTR as a potential marker for human colon cancer. However, our data unexpectedly reveal a protective role of OXTR in both DSS-induced colitis and AOM/DSS-induced CAC tumorigenesis mouse models. Moreover, our analysis of human colitis and CAC samples reveals down-regulation of OXTR expression, indicating complex and context-dependent roles of OXT signaling under these conditions. Furthermore, the down-regulation of OXTR observed in CAC may be mediated by elevated levels of inflammatory cytokines, which are prevalent in chronic inflammation and cancer. Research indicates that tumor necrosis factor-α (TNF-α), a key proinflammatory cytokine, can modulate the expression of numerous genes through activation of the mitogen-activated protein kinase signaling pathway [39]. This pathway impacts various cellular functions including receptor expression [40], which might influence OXTR levels in colonic tissues. Moreover, changes in macrophage polarization, a process influenced by the local cytokine environment, have been shown to affect the expression components of the OXT signaling system in enteric neurons, suggesting that inflammatory states might modulate OXTR expression indirectly through alterations in immune cell function [41]. Notably, we hypothesize that neoplastic lesions in colitis, unlike sporadic colon cancer, may largely operate independently of Wnt signaling, warranting further investigation into the specific function of OXT signaling in Wnt-driven colon cancer [42].

Fucosylation has been established as a crucial modification for maintaining normal colon homeostasis [43], and previous studies have highlighted the protective effects of fucose supplementation through FUT2-mediated fucosylation against colitis onset [12]. In our study, we uncover an alternative mechanism involving B3GNT7-mediated fucosylation, which contributes to colonic mucin formation and is regulated by the intricate gut-brain axis. FUT8, as the sole enzyme responsible for α_1_-6-linked fucosylation [13], adds fucose to the innermost GlcNAc residue of an N-linked glycan. While OXT does not directly affect the mRNA levels of FUTs, our findings suggest that OXT is involved in α_1_-3-linked fucosylation through the non-FUT B3GNT7. Hence, we propose that B3GNT7-mediated α_1_-3-linked fucosylation, not FUT8-mediated α_1_-6-linked fucosylation, plays a crucial role in the maturation of MUC2 protein in the colon. Both these processes are essential for protecting the digestive tract from pathogens and maintaining intestinal health.

In summary, our study provides comprehensive evidence highlighting the crucial role of OXT in maintaining colon homeostasis and protecting against colitis and CAC tumorigenesis. We elucidate the involvement of OXT in regulating intestinal mucin maturation through the fucosylation pathway mediated by B3GNT7. Intriguingly, reduced expression of OXTR and B3GNT7 is observed in patients with colitis and CAC, underscoring the clinical relevance of our findings. Considering the Food and Drug Administration approval and good tolerability of OXT in humans, cotreatment of OXT and l-fucose represents a promising and cost-effective therapeutic avenue for patients suffering from colitis and CAC.

## Materials and Methods

### Experimental animal studies

OXTR^fl/fl^ mice [B6.129(SJL)-Oxtr^tm1.1Wsy^/J, 008471] and villin-cre mice (B6.Cg-Tg[Vil1-cre]997Gum/J, 004586) in the C57BL/6 genetic background were obtained from the Jackson Laboratory. Intestine-specific OXTR null mice (OXTR^△IEC^) were generated by cross-fertilizing the transgenic mice. Sex- and age-matched littermate mice were used for all experiments. The mice were bred and maintained under specific-pathogen-free conditions at the animal facility of the School of Basic Medical Sciences. All animal experiments were conducted following the guidelines of the National Institutes of Health Guide for the Care and Use of Laboratory Animals and were approved by the Scientific Investigation Board of Shandong University (#ECSBMSSDU2019-2-048), Jinan, Shandong Province, China.

### CAC and colitis model

To induce the CAC model, 8-week-old mice were intraperitoneally injected with (10 mg/kg) AOM (Sigma-Aldrich) [25]. After 7 d, 2% DSS (MP Biomedicals) was administered in drinking water for 5 d, followed by regular water for 14 d. This 5-d cycle of 2% DSS was repeated twice, and mice were euthanized after the last DSS cycle. For the AOM-alone-induced colon cancer model, 8-week-old mice were intraperitoneally injected with AOM (10 mg/kg) once a week for 6 weeks, followed by a 5-month waiting period to induce spontaneous colonic carcinogenesis. Mice were euthanized by CO_2_ asphyxiation. For the chronic DSS model, 8-week-old mice were administered 2% DSS in autoclaved drinking water for 6 d, followed by regular water for 5 d. This 5-d cycle of 2% DSS was repeated twice, and mice were sacrificed after the last DSS cycle. For the acute DSS model, 8-week-old mice were administered 2.5% DSS in autoclaved drinking water for 6 d. To investigate the effect of l-fucose [12,44], 200 mM l-fucose (Solarbio) dissolved in saline was given to mice during the DSS administration period. To investigate the effect of OXT, mice were intraperitoneally injected with normal saline or OXT (Cayman Chemical Company, 11799; 1 mg/kg per day) during the DSS administration period [36].

### Pharmacological experiment

LS174T cells were grown in supplemented Dulbecco’s modified Eagle’s medium for 3 d, while organoids were cultured for 7 d in a complete medium to achieve differentiated GCs. Cells were then placed in serum-free media for 16 to 18 h before treatment with inhibitors, including 200 μM 2F-Fuc,1 μmol of L-368,899, 1 μmol of U73122, 1 μmol of nifedipine, 20 μmol of U0126, 1 μmol of PD0325901, or vehicle control for 30 min. Cells were then stimulated with 1 μmol of OXT at the indicated time and concentrations.

### Transfection of siRNA

Cells were transfected with B3gnt7 siRNAs or control siRNAs using INTERFERin (LS174T) or lip3000 (crypt) according to the manufacturer’s instructions. The targeted siRNA sequences were designed and synthesized by Generay Biotechnology (Shanghai).

### Analysis of human patient datasets and human cohort

Gene expression data correlation analysis in colonic mucosal biopsies from human patients with colitis and patients with cancer was obtained from GEO databases GSE37283 and GSE47908. Colonic tissue biopsies were obtained from Qilu Hospital of Shandong University, and informed consent was obtained from all patients. The use of human tissues was approved by the Medical Institutional Ethics Committee of Qilu Hospital, Shandong University, China (#KYLL-202210-049).

### Statistical analysis

The statistical significance of group differences was assessed using GraphPad Prism 8.0 (GraphPad Software). Data are presented as the means ± SEM and based on experiments performed at least in triplicate. All *n* numbers are biological replicates, and all Western blots are representative picture in the whole article. The 2-way analysis of variance (ANOVA) test was used for the comparison between the 2 samples, and the unpaired Student’s *t* test was used for the comparison between the 2 groups. A *P* value of less than 0.05 was considered statistically significant.

## Data Availability

All data needed to evaluate the conclusions in the paper are present in the paper and/or the Supplementary Materials. The RNA sequencing data were uploaded to GEO with the accession code PRJNA996718. The GEO accession codes for previously published datasets used in this study are GSE37283, GSE1113002, and GSE47908.
