# Supplementary material for: Oxytocin Alleviates Colitis and Colitis-Associated Colorectal Tumorigenesis via Noncanonical Fucosylation
Source: Research (Wash D C). 2024 Jul 8;7:0407. doi: 10.34133/research.0407 (PMC11228076; doi:10.34133/research.0407)
Supplement: Supplementary 1 — Supplementary Methods Figs. S1 to S9 Tables S1 to S4 [file research.0407.f1.zip › supplement-research (with Highlighted Changes).docx]

**Oxytocin Alleviates Colitis and Colitis-associated Colorectal Tumorigenesis via Non-Canonical Fucosylation**

Xia Wang,^1,2,3,9^ Dawei Chen,^4,8,9^ Mengnan Guo,^4^ Yao Ning,^4^ Mingze Geng,^4^ Jing Guo,^1,2,3^ Jiahui Gao,^1,2,3^ Dong Zhao,^1,2,3^ Yupeng Zhang,^6^ Qianpeng Li,^7^ Lixiang Li,^1,2,3^ Shiyang Li,^1,3,5^ Yanqing Li,^1,2,3^ Xiaoran Xie,^1,2,3*^ Xiuli Zuo,^1,2,3*^ Jingxin Li^4*^

1. Department of Gastroenterology, Qilu Hospital of Shandong University, Jinan, Shandong, 250012, China
2. Laboratory of Translational Gastroenterology, Qilu Hospital of Shandong University, Jinan, Shandong, 250012, China
3. Shandong Provincial Clinical Research Center for digestive disease, Jinan, 250012, China
4. Department of Physiology, School of Basic Medical Sciences, Cheeloo College of Medicine, Shandong University, Jinan, Shandong, 250012, China
5. Advanced Medical Research Institute, Shandong University, Jinan 250012, China.
6. Department of Molecular Plant Biology, Norwegian Institute of Bioeconomy Research, Ås 1430, Norway
7. Department of Hematology, Weifang People's Hospital, Weifang, Shandong, 261000, China.
8. Present address: Institute of Clinical Molecular Biology, Christian-Albrechts-University Kiel and University Medical Center Schleswig-Holstein, Kiel, 24105, Germany
9. These authors contributed equally.

* Correspondence: X. X. (xiaoran_xie@sdu.edu.cn), X. Z. (zuoxiuli@sdu.edu.cn) or J. L. (ljingxin@sdu.edu.cn)

**Supplementary methods**

Disease Activity Index (DAI) Measurement:

To quantify the severity of induced colitis, including body weight loss, stool consistency, and bleeding, the Disease Activity Index (DAI) was used. DAI is the average score of body weight loss (0, none; 1, 1–5%; 2, 5–10%; 3, 10–15%; 4, > 15%), stool consistency (0, normal; 1 and 2, loose stool; 3 and 4, diarrhea), and stool bleeding (0, negative; 1, +; 2, ++; 3, +++; 4, ++++), recorded from day 0 to 7. After the mice were sacrificed, the colon was removed, and its length was measured from the ileocecal junction to the anal verge. The proximal rectal colon was placed in 4% paraformaldehyde for histology, and the distal parts were kept in an RNA fixer (Aidlab) for later RNA isolation.

Isolation of Intestinal Epithelial Cells (IECs):

Mouse intestine tissues were isolated, opened longitudinally, washed in 0.04% sodium hypochlorite, and then shaken in HBSS containing 5% FBS, (5 mM) EDTA, and (1 mM) dithiothreitol for 30 minutes. The remaining tissue was discarded, and IECs in the supernatant were collected by centrifugation.

Sample Collection, Histology, and Immunostaining:

Intestinal tissues from the jejunum were surgically removed from euthanized mice and flush-cleaned using PBS. The tissues were fixed in 4% paraformaldehyde, paraffin-embedded, and sectioned at 4 μm thickness for hematoxylin and eosin (H&E) staining and immunostaining. Antigen retrieval was performed using sodium citrate buffer, then was permeabilized with a solution containing 1% Triton X-100, 4% donkey serum, and 1% BSA. tissue sections were incubated overnight at 4°C with anti-OXTR and anti-MUC2 antibodies. After incubation with secondary antibodies for 2 h at room temperature, DAB staining and Hematoxylin counterstaining were used for immunohistochemistry, while DAPI was used for nucleus staining in immunofluorescence. The quantifications of OXTR-positive area measured by image J. Briefly, after converting the picture format to “RGB Stack”, the image with the highest agreement with the positive area is marked in the “Threshold” interface, and the mean gray value was measured.

Alcian Blue-Periodic Acid Schiff’s (AB-PAS) Staining:

AB-PAS staining was performed to detect acidic mucin in sections from paraffin-embedded tissue using a commercial AB-PAS kit. The sections were subjected to periodic acid and Schiff Reagent treatment to stain for acidic mucin.

Measurement of Mucus Layer Thickness:

Colon tissue from OXTR^fl/fl^ and OXTR^△IEC^ mice was isolated and fixed in methanol-Carnoy. The thickness of the mucus layer was measured in distal colonic tissue sections using image-pro Plus software in both PAS- and PAS-AB-stained sections. Twenty different measurements were performed in two different tissue sections per stain in the distal colon of each mouse. The mucus layer thickness was evaluated in all samples that presented fecal pellets.

RNA Extraction and Real-Time PCR Analysis:

Total RNA was extracted from tissue or cells using an EASYspin Plus kit (Aidlab), and cDNA was synthesized using the QuantiTect Rev. Transcription Kit (Vazyme, Nanjing). Real-time PCR was performed using SYBR Green qPCR Mix (Vazyme, Nanjing) on a Bioer-Lightcycler. Relative gene expression was calculated using the △△Ct method and normalized to GAPDH. The primers used in the present study are listed in Supplementary Table 1, and synthesized by the Beijing Genomics Institute (Beijing, China).

RNA-seq:

RNA-seq was performed by Majorbio company(PRJNA996718). Total RNA was extracted from tissue using TRIzol® Reagent and genomic DNA was removed using DNase I. RNA-seq transcriptome library was prepared using 1 μg of total RNA. The raw paired-end reads were trimmed, and quality controlled. Clean reads were aligned to the reference genome using HISAT2 software, and the mapped reads were assembled by StringTie. Relevant differential genes have been listed in Supplementary Table 3.

Protein Extraction and Western Blotting:

Intestinal tissues, tumor tissues, or organoid samples from each group were lysed and subjected to SDS-PAGE. The proteins were transferred to a PVDF membrane and probed with specific primary antibodies. Detection was performed using HRP-conjugated secondary antibodies or streptavidin, followed by chemiluminescence. For the MUC2 blot, a large protein with many disulfide bonds, dithiothreitol (DTT), beta-mercaptoethanol (β-ME), and sodium dodecyl sulfate (SDS) are added during sample preparation. In addition, 8% acrylamide concentration and 2-hour electrophoresis time were used to help MUC2 migrate. The antibodies used in this study are listed in Supplementary Table 2.

Transmission Electron Microscopy (TEM) Analysis:

Intestinal sections were fixed in fixative for TEM at 4°C and post-fixed with 1% OsO4 for 2 hours. After embedding in resin, ultrathin sections (70 nm) were cut and stained. Colonic section structures were analyzed using a Hitachi HT-7800 TEM.

Mouse Colonic Crypts Isolation:

Mouse crypt isolation, organoid development, and passage were performed as previously described. The colon segment was cut lengthwise, washed with DPBS, and cut into 2 mm pieces. The pieces were transferred to a 50 mL conical tube with cold DPBS and pipetted up and down three times. After rinsing, the tissue pieces were incubated in 15 mL of (2.5 mM) EDTA at 4°C for 1 hour. The tissue pieces were then resuspended in cold DPBS, and the supernatant containing isolated crypts was collected. Freshly isolated crypts were cultured using the Mouse Colonic Organoid Kit from bioGenousTM and plated in Matrigel in 48-well plates.

**Supplementary figures and figure legends**

**Figure S1. Expression of OXTR in Intestinal Epithelium.**

**(A)** Representative images of OXTR IHC staining in WT (left) and CAC (right) tissue. Scale bar, 100 μm. (n=3) **(B)** Immunofluorescence staining of nuclei (DAPI, blue) and OXTR (green) in OXTR^fl/fl^ (up) and OXTR^△IEC^ (down) mice. scale bar, 100 μm. (n=3) **(C)** Immunofluorescence staining of nuclei (DAPI, blue), MUC2 (red), and OXTR (green). scale bar, 100 μm. (n=3)

**Figure S2. IECs ablation of OXTR does not affect CRC.**

OXTR^fl/fl^ and OXTR^△IEC^  mice were treated with 5 cycles of AOM to induce a colitis-independent CRC model. **(A)** Flow chart of the induction procedure. **(B)** Representative photos of the colons. **(C)** Colon length. (n=8) **(D)** Number of tumors in the colon. (n=8) **(E)** The percentage of tumors by different diameters. (n=8) **(F)** Statistical analysis of spleen weight. (n=8) **(G)** Representative H&E-stained colon sections. Scale bar, 1mm. **(H)** Relative inflammatory cytokine mRNA levels in the colon were measured by RT-qPCR. (n=8) Data are presented as the mean ± SEM. n.s, no significance. P-values were determined using C, D, F) unpaired two-tailed Student’s t-test or H) two-way analysis of variance (ANOVA).

**Figure S3. The absence of OXTR does not Trigger intestinal inflammation in a steady state.**

**(A)** Representative photos of the colons. **(B)** Colon length. (n=3) **(C)** Spleen weight. (n=3) **(D)** Representative H&E-stained of colon sections. (n=3) Scale bar, 100 μm. **(E)** Cytokine production in whole cells from the colon. (n=3) Data are presented as the mean ± SEM. *P < 0.5, **P < 0.1, ***P < 0.01. P-values were determined using B, C) unpaired two-tailed Student’s t-test, or E) two-way analysis of variance (ANOVA).

**Figure S4. Mucus barrier-related gene expression in OXTR^fl/fl^ and OXTR^△IEC^ murine colitis.**

1. **H)** RT-qPCR analysis of the relative expression of *Oxtr, Cldn2, Tjp1, Cldn5, Occlaudin, Muc2, Manf,* and *Tff3* in mice was treated with or without DSS. Vehicle + OXTR^fl/fl^ (n=4), Vehicle + OXTR^△IEC^ (n=8), DSS + OXTR^fl/fl^ (n=8), DSS + OXTR^△IEC^ (n=4). Data are presented as the mean ± SEM. n.s, no significance. P-values were determined by using a two-way analysis of variance (ANOVA).

**Figure S5. OXTR is important for preserving inner mucus layer integrity following challenges with DSS.**

1. **B)** Western blot analysis of the barrier proteins level in the IECs from OXTR^fl/fl^ and OXTR^△IEC^ mice treated with or without 2.5% DSS. (n=5) **(C)** Transmission electron microscopy of colons obtained from untreated mice or mice treated with 2.5% DSS. Scale bar, 500 nm. (n=3) **(D)** Representative micrographs of periodic acid-Schiff (PAS)-AB staining. Scale bar, 100 μm (n=3) **(E)** Quantifications of mucus thickness in colon sections with contents from vehicle- and DSS-treated mice. (n=3) **(F)** Representative micrographs of periodic acid-Schiff (PAS) staining. Scale bar, 100 μm. (n=3) **(G)** The number of PAS-positive cells was counted randomly in 7 crypts for each mouse. Histogram demonstrating the number of Goblet cells per crypt. (n=3). **(H)** Immunofluorescence staining of Muc2 (red) and nuclei (DAPI, blue), scale bar, 100 μm. (n=3) **(I)** The number of MUC2-positive cells was counted randomly in 5 crypts for each mouse. (n=3) Data are presented as the Mean ± SEM. n.s, no significant, **P < 0.1, ***P < 0.01. P-values were determined by using a two-way analysis of variance (ANOVA).

**Figure S6. OXT promotes MUC2 and B3GNT7 expression in vitro.**

**(A and B)** Western blot analysis of the MUC2 level in LS174T cells **(A)** and colonic organoids **(B)** treated with OXT (1μmol) or L-368,899 (1μmol). (n=5). **(C)** Immunofluorescence staining of Muc2 (red) and nuclei (DAPI, blue) in colonic organoids. (n=5). Scale bar, 100 μm. **(D-K)** RT-qPCR **(D, E, H, J)** and Western blot analysis **(F, J, I, K)** of B3GNT7 in LS174T cells **(D, F, H, I)** (n=5) and colonic organoids (E, G, J, K) (n=5). Data are presented as the mean ± SEM. *P < 0.5, **P < 0.1, ***P < 0.01. P-values were determined by using an unpaired two-tailed Student’s t-test.

**Figure S7. Fucosyltransferase expression in OXTR^fl/fl^ and OXTR^△IEC^ murine colitis.**

**(A)** The heatmap of 10 glycosyltransferases (B3gnts) and 10 fucosyltransferases (FUTs) mRNA expression in OXTR^fl/fl^ (n=3) and OXTR^△IEC^ (n=3) mice were treated with or without 2.5%DSS. The RNA-Seq gene count data was normalized using DESeq2 variance-stabilizing transformation. **(B and C)** Relative FUTs mRNA levels in mice treated with (n=8) or without DSS (n=6) were measured by RT-qPCR. **(D)** RT-qPCR analysis of the relative expression of FUTs in human normal or colitis tissue (n=4). Data are presented as the mean ± SEM. n.s, no significance. P-values were determined by using a two-way analysis of variance (ANOVA).

**Figure S8. Oxytocin treatment reduces colon tumor burden.**

OXTR^fl/fl^ mice were treated with AOM and 3 cycles of 2% DSS (n=4) or DSS + OXT (n=5) to induce inflammation-driven colorectal cancer. **(A)** Flow chart of the induction procedure. **(B)** Representative photos of the colons. **(C)** Colon length. **(D)** Number of tumors in the colon. **(E)** The percentage of tumors by different diameters. **(F)** Statistical analysis of spleen weight. **(G)** Survival curve assessed by log-rank (Mantel-Cox) test. **(H)** Representative H&E-stained colon sections. Scale bar, 1mm. **(I)** Relative inflammatory cytokine mRNA levels in the colon were measured by real-time polymerase chain reaction. **(J, K)** Western blot analysis of the LTL, MUC2, and B3GNT7 levels in mice. (n=5) **(L)** Representative micrographs of periodic acid-Schiff (PAS)-AB staining. Scale bar, 100 μm. Data are presented as the mean ± SEM. *P < 0.5, **P < 0.1, ***P < 0.01. P-values were determined using C, D, F) unpaired two-tailed Student’s t-test or G) two-way analysis of variance (ANOVA).

**Figure S9. The quantification of the Western blots.**

(A-B) Quantitative analyses of protein expression in FigureS5A and FigureS5B. (C-D) Quantitative analyses of MUC2 expression in FigureS6A and FigureS6B. (E-J) Quantitative analyses of B3GNT7 expression in Figure3E, Figure3F, FigureS6F, FigureS6G, FigureS6I, and FigureS6K. (K-L) Quantitative analyses of MUC2 expression in Figure3I and Figure3J. (M-N) Quantitative analyses of B3GNT7 expression in Figure3K and Figure3M. (O) Quantitative analyses of LTL expression in Figure4A. (P-Q) Quantitative analyses of MUC2 expression in Figure4D and Figure4E. (R) Quantitative analyses of LTL expression in Figure4F. (S-T) Quantitative analyses of MUC2 and B3GNT7 expression in Figure4Q. (U-V) Quantitative analyses of protein expression in FigureS8J and FigureS8K. (W) Quantitative analyses of B3GNT7 expression in Figure5J. (X) Quantitative analyses of protein expression in Figure6E. (Y) Quantitative analyses of MUC2 expression in Figure6H. Data are presented as the mean ± SEM. *P < 0.5, **P < 0.1, ***P < 0.01. P-values were determined using A, B, D, K, L, P, Q, S, T, U, V, X) two-way analysis of variance or C, E, F, I, J, O, Y) unpaired two-tailed Student’s t-test or G, H, M, N, R, W) two-way analysis of variance (ANOVA).
