# Supplementary figures and images for: Oxytocin Alleviates Colitis and Colitis-Associated Colorectal Tumorigenesis via Noncanonical Fucosylation
Source: Research (Wash D C). 2024 Jul 8;7:0407. doi: 10.34133/research.0407 (PMC11228076; doi:10.34133/research.0407)

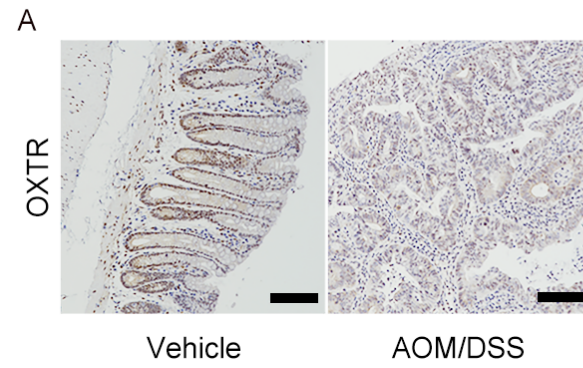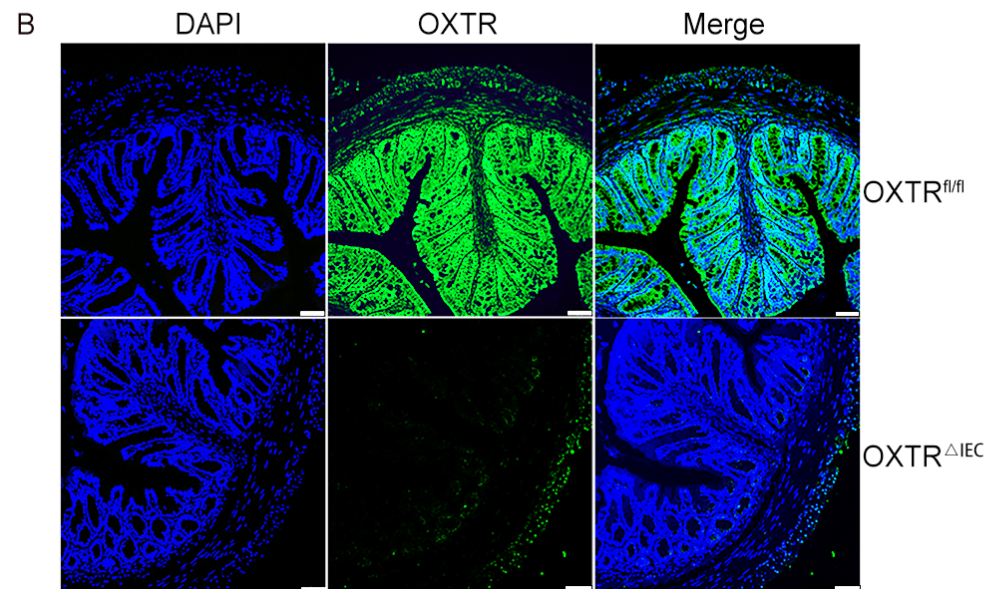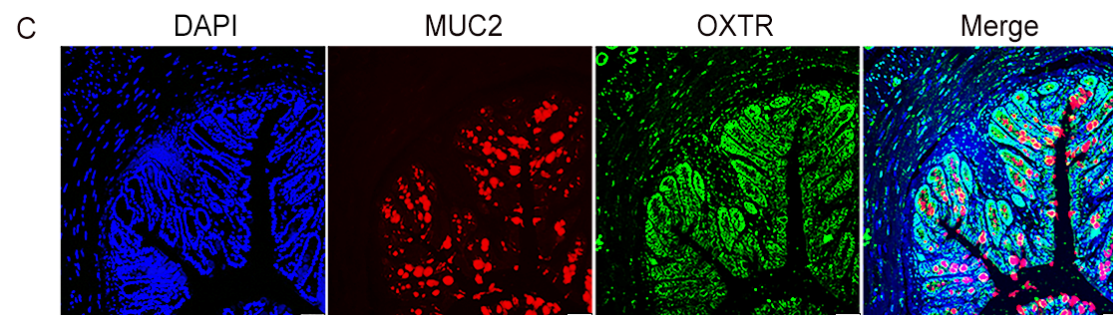

Supplement: Supplementary 1 — Supplementary Methods Figs. S1 to S9 Tables S1 to S4 [file research.0407.f1.zip › FIG S1.pdf]

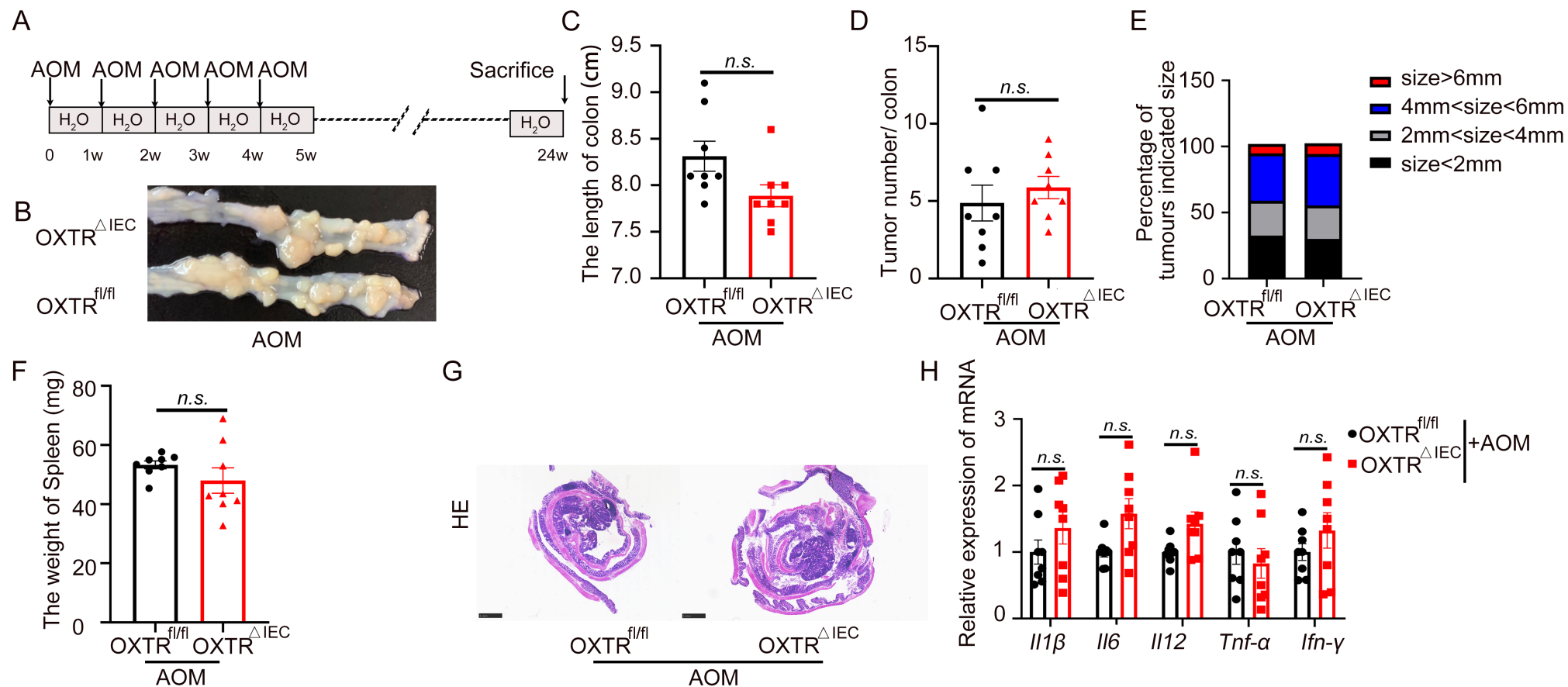

Supplement: Supplementary 1 — Supplementary Methods Figs. S1 to S9 Tables S1 to S4 [file research.0407.f1.zip › fig S2.pdf]

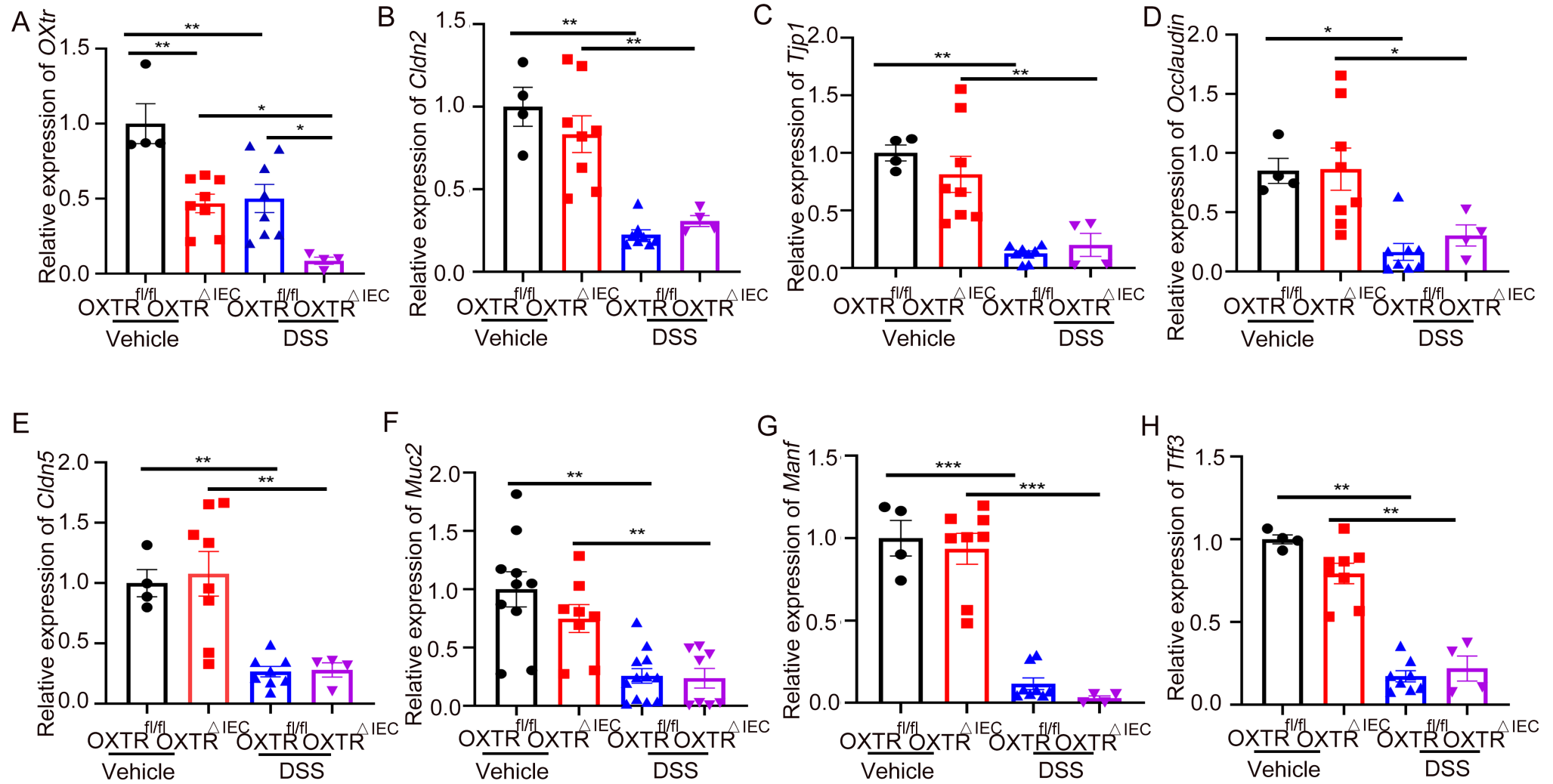

Supplement: Supplementary 1 — Supplementary Methods Figs. S1 to S9 Tables S1 to S4 [file research.0407.f1.zip › fig S4.pdf]

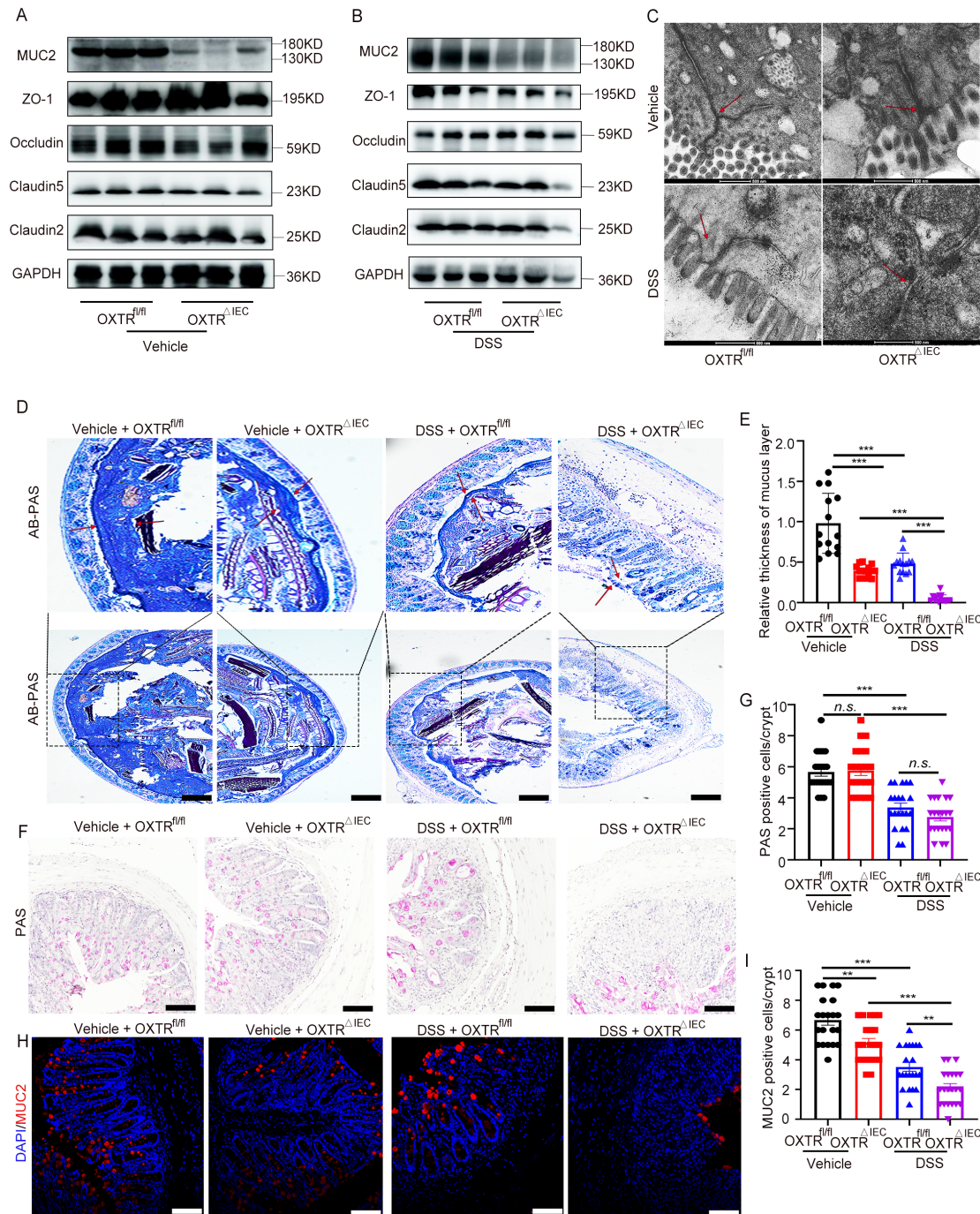

Supplement: Supplementary 1 — Supplementary Methods Figs. S1 to S9 Tables S1 to S4 [file research.0407.f1.zip › fig S5.pdf]

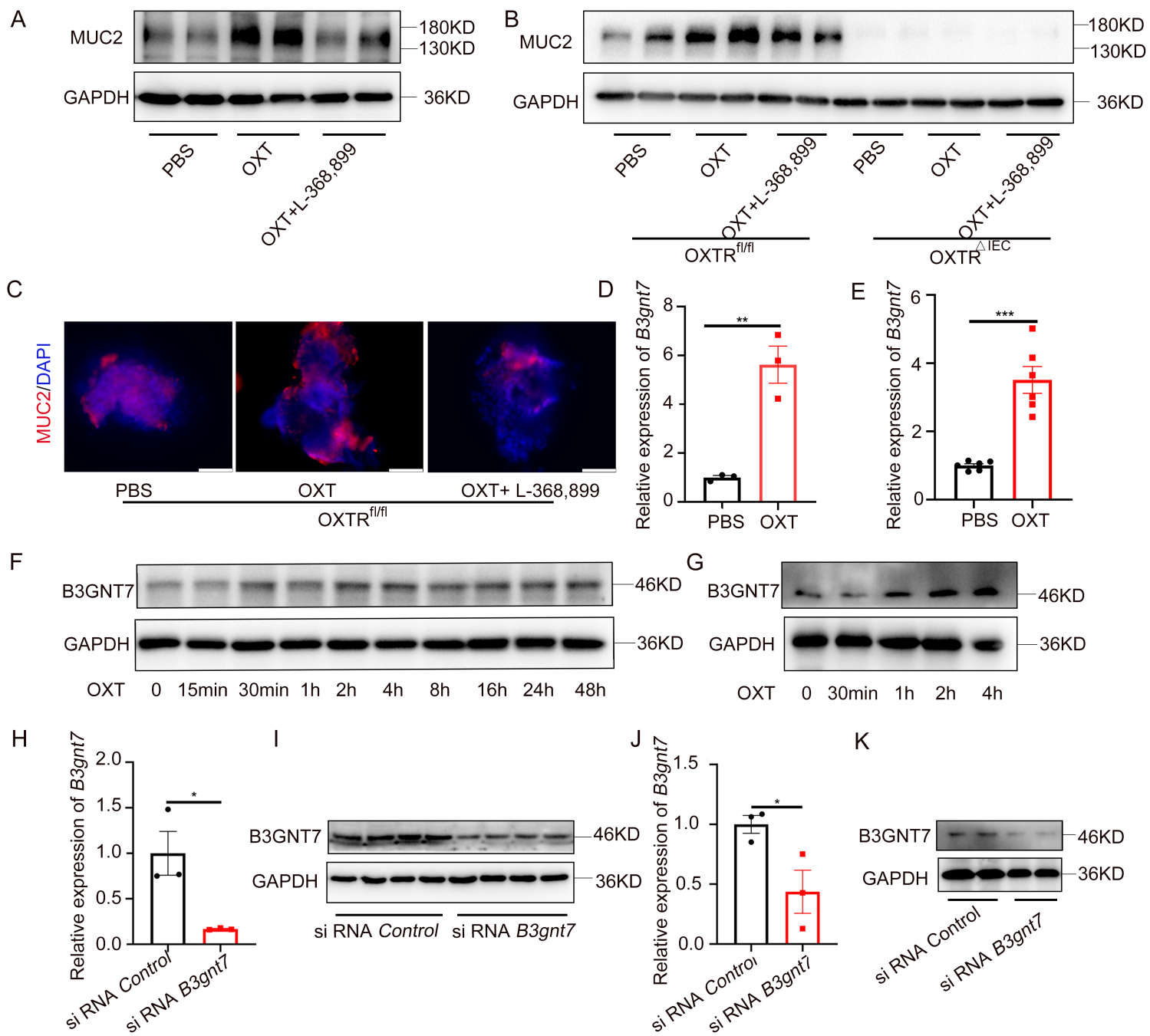

Supplement: Supplementary 1 — Supplementary Methods Figs. S1 to S9 Tables S1 to S4 [file research.0407.f1.zip › fig S6.pdf]

A

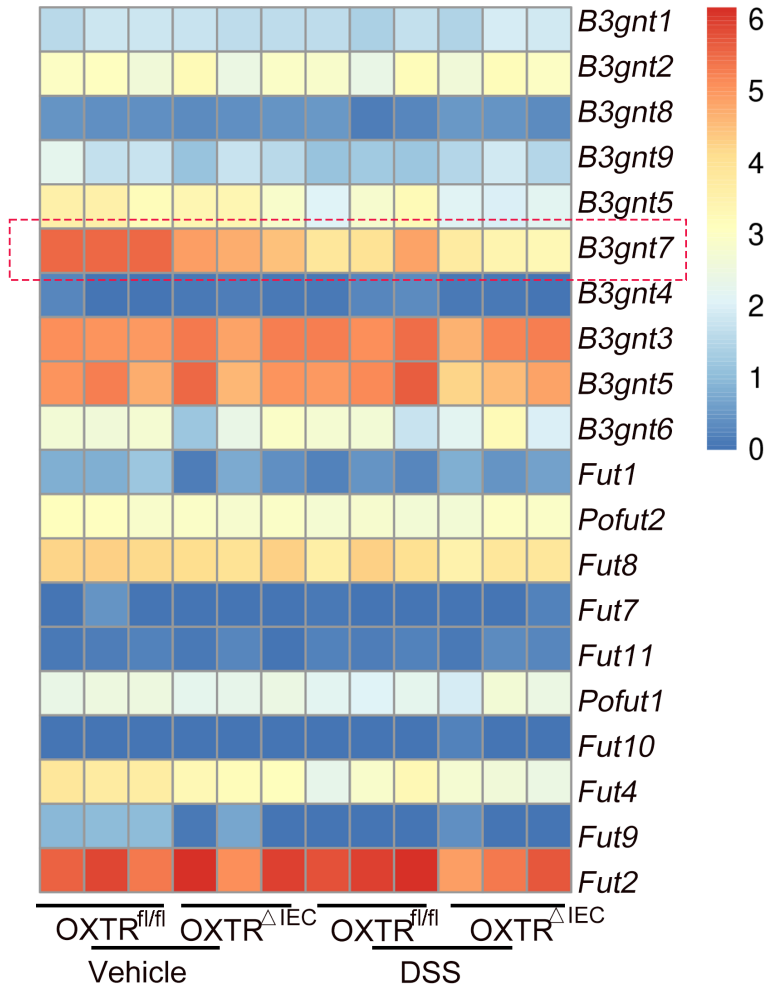

B

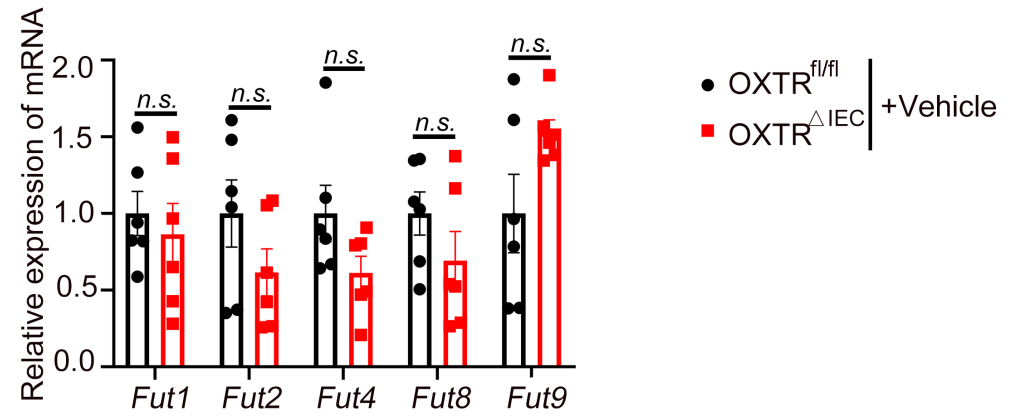

C

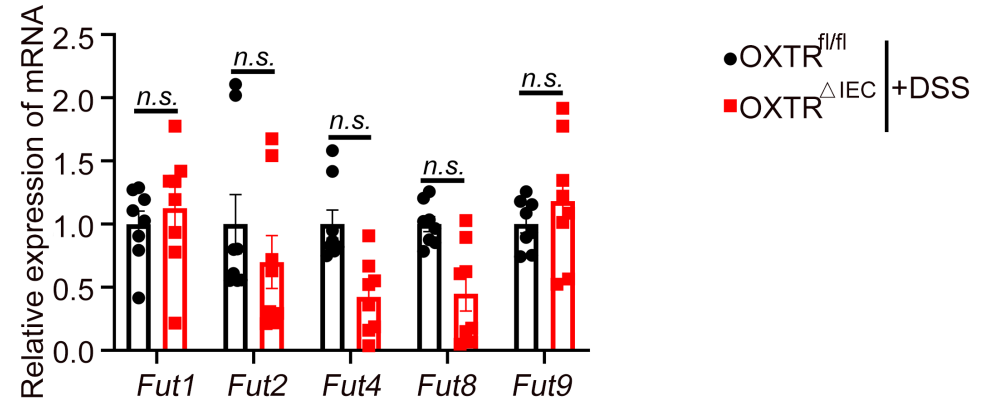

D

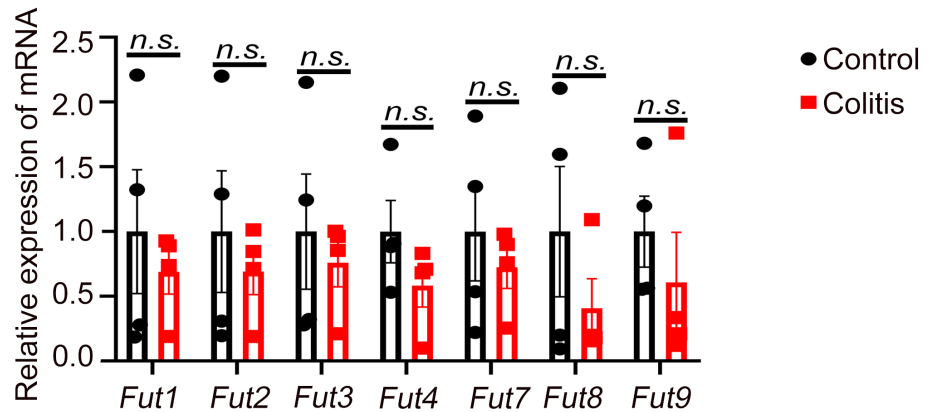

Supplement: Supplementary 1 — Supplementary Methods Figs. S1 to S9 Tables S1 to S4 [file research.0407.f1.zip › fig S7.pdf]

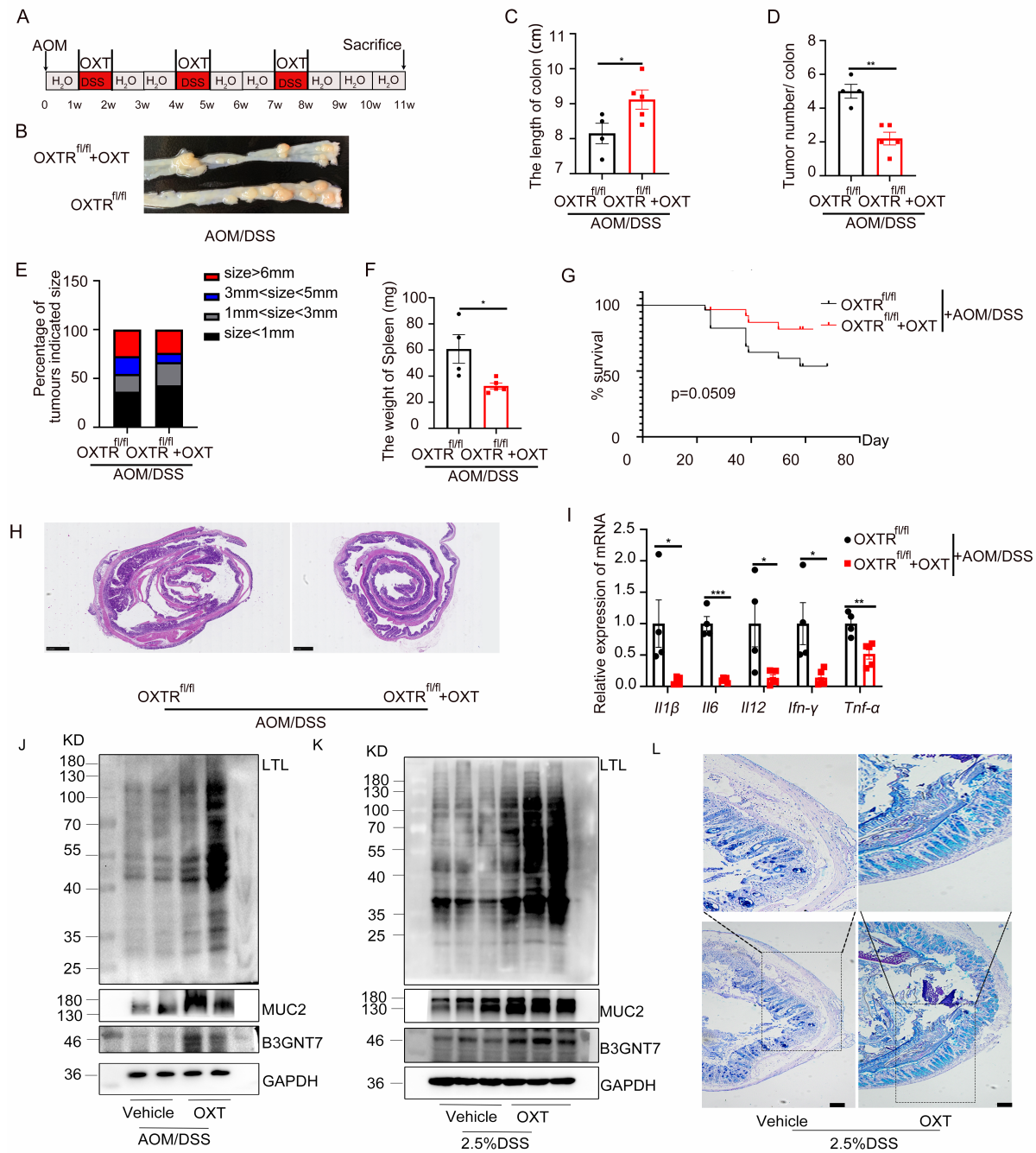

Supplement: Supplementary 1 — Supplementary Methods Figs. S1 to S9 Tables S1 to S4 [file research.0407.f1.zip › fig S8.pdf]

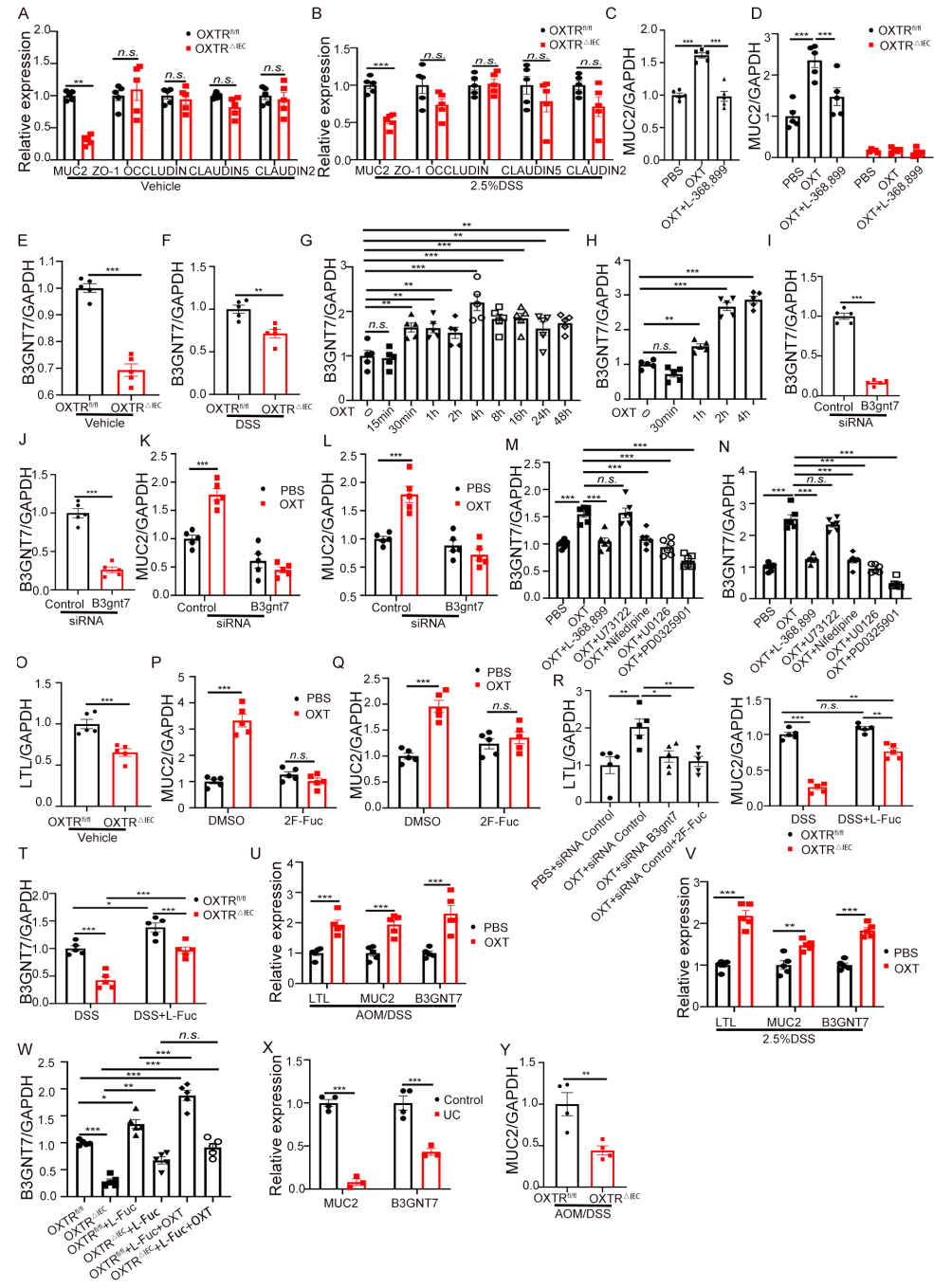

Supplement: Supplementary 1 — Supplementary Methods Figs. S1 to S9 Tables S1 to S4 [file research.0407.f1.zip › FigS9.pdf]

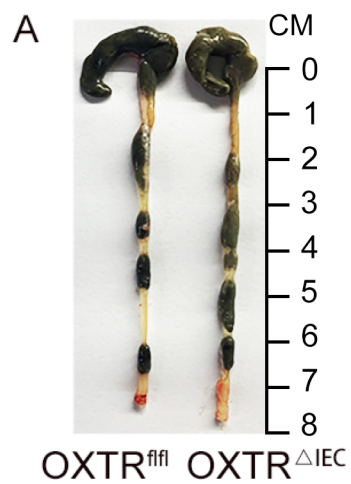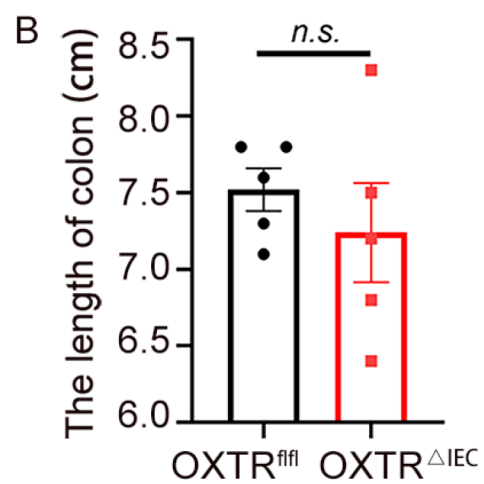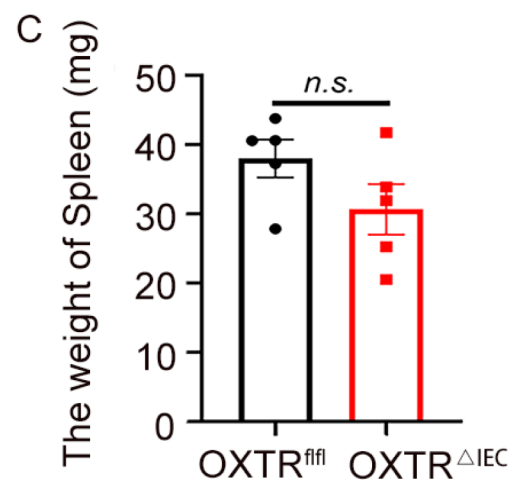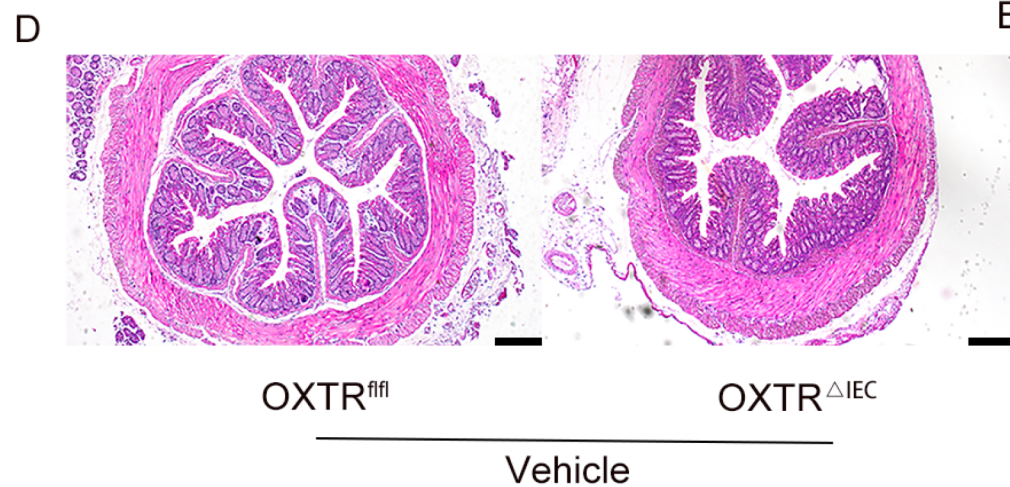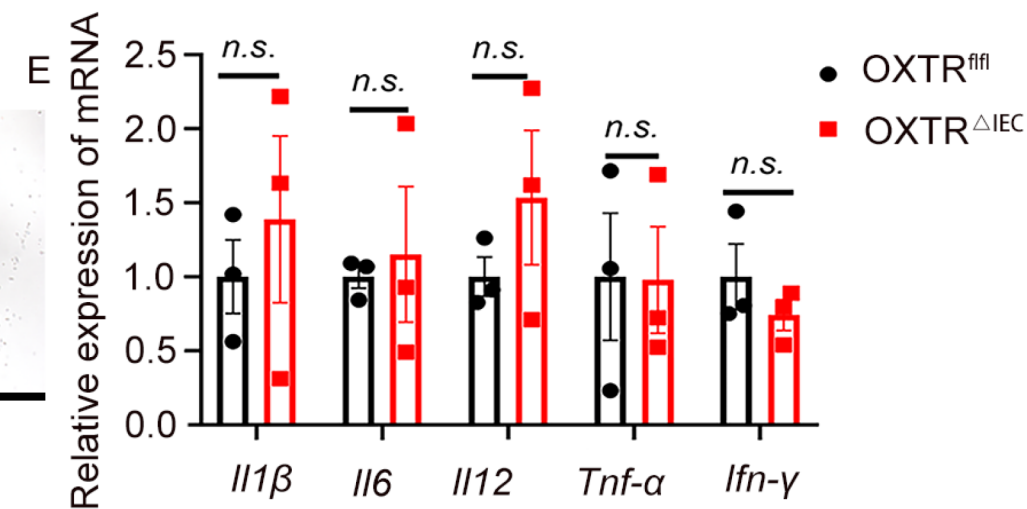

Supplement: Supplementary 1 — Supplementary Methods Figs. S1 to S9 Tables S1 to S4 [file research.0407.f1.zip › renamed_056a4.pdf]
